# Protein-Ligand Blind Docking Using QuickVina-W With Inter-Process Spatio-Temporal Integration

**DOI:** 10.1038/s41598-017-15571-7

**Published:** 2017-11-13

**Authors:** Nafisa M. Hassan, Amr A. Alhossary, Yuguang Mu, Chee-Keong Kwoh

**Affiliations:** 1 School of Biological Sciences, Nanyang Technological University, Singapore, Singapore; 2 School of Computer Science and Engineering, Nanyang Technological University, Singapore, Singapore

## Abstract

“Virtual Screening” is a common step of in silico drug design, where researchers screen a large library of small molecules (ligands) for interesting hits, in a process known as “Docking”. However, docking is a computationally intensive and time-consuming process, usually restricted to small size binding sites (pockets) and small number of interacting residues. When the target site is not known (blind docking), researchers split the docking box into multiple boxes, or repeat the search several times using different seeds, and then merge the results manually. Otherwise, the search time becomes impractically long. In this research, we studied the relation between the search progression and Average Sum of Proximity relative Frequencies (ASoF) of searching threads, which is closely related to the search speed and accuracy. A new inter-process spatio-temporal integration method is employed in Quick Vina 2, resulting in a new docking tool, QuickVina-W, a suitable tool for “blind docking”, (not limited in search space size or number of residues). QuickVina-W is faster than Quick Vina 2, yet better than AutoDock Vina. It should allow researchers to screen huge ligand libraries virtually, in practically short time and with high accuracy without the need to define a target pocket beforehand.

## Introduction

In the in silico drug discovery domain, “Virtual Screening” is defined as “automatically evaluating very large libraries of compounds using computer programs”^1^. It is entitled to produce and screen drug candidates more effectively than the physical assessment of thousands of diverse compounds a day, using high-throughput screening robotics, and thus increasing the rate of drug discovery while reducing the need for expensive laboratory work. Molecular docking is the core of virtual screening. It aims at prediction of the modes and affinities of non-covalent binding between a pair of molecules. Oftentimes, the molecules consist of a macromolecule (the receptor) and a small molecule (the ligand). The multidimensional search space of the ligand includes the degrees of freedom of its translation, rotation, and torsions of flexible bonds that may exist within it. Some packages consider flexibility in the receptor as well^2–4^.

A successful docking application needs to have two pillars: 1) a method to explore the ligand-receptor conformation space for plausible poses [the search algorithm], and 2) a method to relatively order those plausible poses [the scoring function]. In a recent study, Wang *et al*. performed a comprehensive evaluation of ten famous currently available docking programs, including five commercial and five academic programs. Wang *et al*. studied their accuracies of binding pose prediction (sampling power) and their binding affinity estimation (scoring power) and concluded that AutoDock Vina^4^ has the highest scoring power among them^5^.

AutoDock Vina (referred to as Vina hereinafter) utilizes a powerful hybrid scoring function (empirical + knowledge-based) and employs an evolutionary search, for the minimum-energy docking conformations (solutions). In evolutionary search, a solution is iteratively optimized until a considerably accepted solution is found. If we think of every “possible solution” as a “point in the search space”, the search process in Vina is performed as iterations of 1) global optimization in the form of modified Monte Carlo^6^, performed on initial seeds (pseudorandom points), followed by 2) local optimization with BFGS method^7^. The modified Monte Carlo search is to perform a cycle of what we call an “essential” local optimization first before testing the proposed point according to the Metropolis acceptance criteria. Please refer to the supplementary material for illustration on the search process. The dimensions of the search space in Vina family include three translations and three rotations of the ligand (applied at its root), as well as the torsion angles of all active (rotatable) bonds within it or within the receptor. That is to say, the number of degrees of freedom (N) in Vina is 6 + number of rotatable bonds. Quick Vina (referred to as ‘QVina 1’ hereinafter)^8^ was developed to speed up Vina using heuristic to save local optimization by trying the potentially significant points only. A “potentially significant point” is a point that is expected to undergo optimization through a new pathway not explored by other points before. The technique is to check any provisional point against the search thread history of visited points and accept only the points where there is at least one (near) history point such that for each design variable pair (provisional point – history point variable pair), the partial derivatives of the scoring function with respect to the variable at both points either have opposite signs or one of them is zero. This means that accepted points are assured to be in a new unexplored energy well. QVina 1 uses less search time than the original Vina does, however it was designed to run on impractically big number of CPUs to overcome the high rate of false negatives. QuickVina 2 (referred to as ‘QVina 2′ hereinafter)^9^ restored the lost accuracy of QVina 1 (compared to Vina) by using a more robust test that considers the *first-order-consistency-check*. Please refer to the methodology and supplementary methodology sections of our previous work^9^ for more details and illustrations. Vina, QuickVina 1 and 2, depend on multiprocessing to achieve fast search, where several threads traverse the search space simultaneously.

Blind Docking refers to docking a ligand to the whole surface of a protein without any prior knowledge of the target pocket. Blind docking involves several trials/runs and several energy calculations before a favorable protein-ligand complex pose is found. However, the number of trials and energy evaluations necessary for a blind docking job is unknown. In their paper, Hetenyi, and Van Der Spoel recommended a number of trials to exceed 100 times, and at least 10 million energy evaluations per trial in case of flexible ligands^10^.

When it comes to Blind Docking, most -if not all- of the famous (non-exhaustive) docking tools are quite limited. That is because the stochastic nature of search for a fixed number of steps makes it unlikely to sample the whole energy landscape surface thoroughly enough to find all the important poses. Researchers usually mitigate this issue by either reducing the search complexity (by splitting the docking box into multiple boxes^11,12^ or sacrificing the flexibility of some parts of the ligand^13^), or repeating the search several times using different seeds^14^, and in both cases, they later merge the results together manually.

In this work, we show that enabling inter-process communication would enhance both the accuracy and the speed of the decision-making process, and that such enhancement is directly proportional to the amount of the accumulated common wisdom, among all the threads. Based on that, we introduced a new docking tool, QuickVina-W (referred to as QVina-W herein after), that is suitable for blind docking, eliminating the need to run the docking tool several times or to split the docking box and then to merge the search results.

## Methods and Materials

### Theory

The philosophy behind QuickVina^8,9^ is to optimize local searches (the most time-consuming search step) only to potentially significant points, by means of keeping track of the visited points in the search history and examining every new potential point against up to P history points before it is accepted and allowed to undergo local optimization. This was perfect for relatively small search spaces. However, it is quite limited for large-sized search space, because the search threads are diluted over the huge search volume, and hence inefficient sampling takes place.

The core of enabling wide docking box search is substituting the first few checks against a thread history with checks on a high-quality collection of common history points. That is to say, the normal (P) checks in QVina is split into two steps: The first step, Global step (G), is to check a small number P_1_ (≪P) of high quality points from all available threads history. The second step, Individual step (I), is the normal QVina 2 check against thread’s individual history points P_2_ (=P − P_1_). This way,

- Having history from *other* threads allows us to make use of other threads experience and make decisions in *already explored* energy landscape areas, while having history from an *individual* same thread allows us to make decisions in *virgin* areas.
- For a ***significant*** point, starting with P_1_ check decreases the number of checks needed before accepting the point (increased decision-making speed).
- For an ***insignificant*** potential point on the other hand, having the number of high quality checks, P_1_ kept to a considerably small value, leaving *large enough* number of history points to check in P_2_ before rejecting a point, ensures *confidence* that this rejection is not due to a false negative (no compromise in accuracy). Going through a full set of P checks then rejecting a potential insignificant point is faster than sending it to a set of unnecessary iterations of local optimization, the most time-consuming step of the search (increased search speed).
- The more the time passes, the more the high-quality points accumulate in the global history, the faster (and the more accurate) the decision is taken in the G stage. This will end up with each thread being either thoroughly exploring unexplored areas or just traversing explored areas.
- The increased speed allowed us to scan more points in the same time interval, increasing the overall search accuracy of the tool.

**Figure 1:**
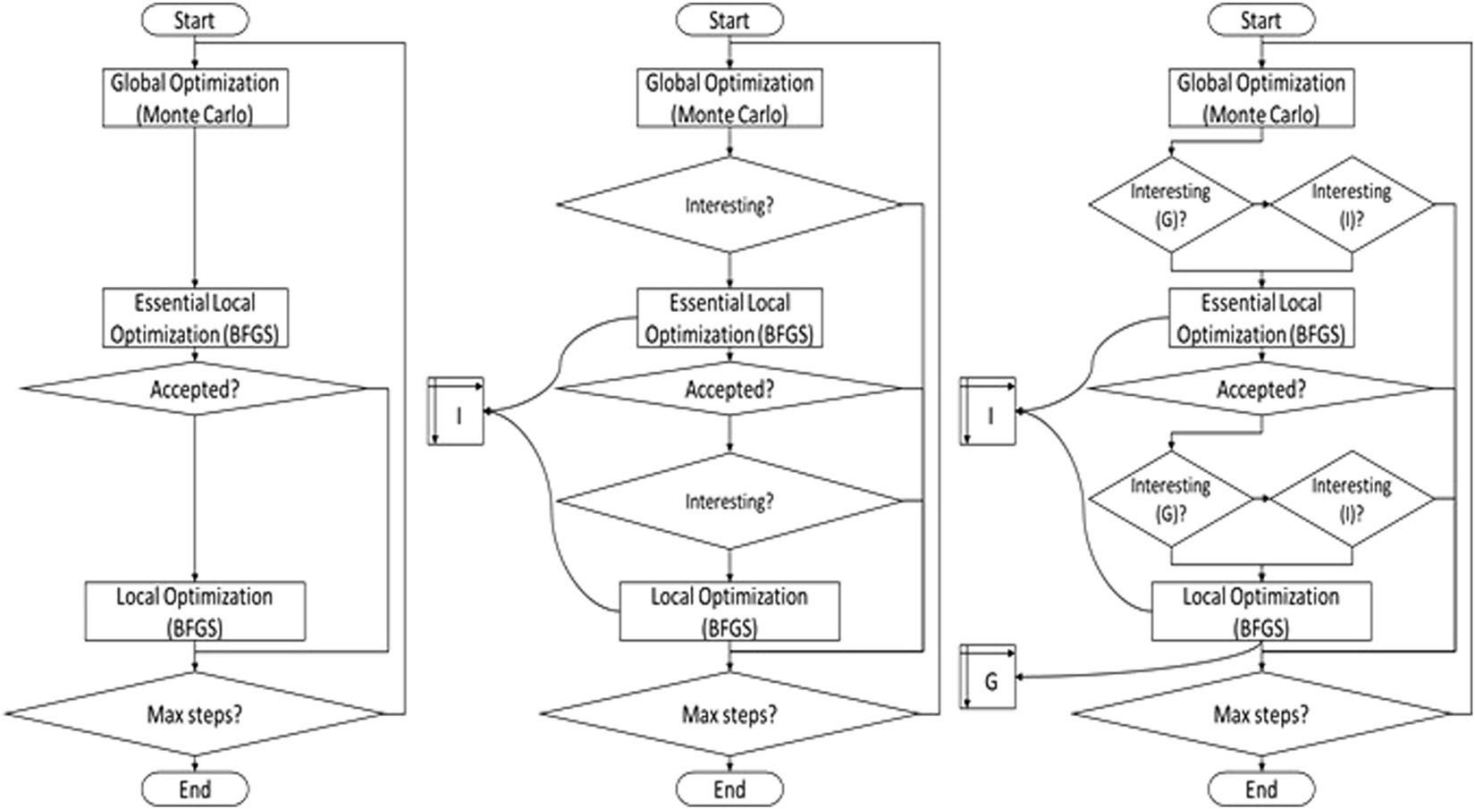
Flow chart of the search algorithm. Flow chart of the search algorithm in Vina, QVina 2, and QVina-W respectively from left to right.

In Fig. 1, we show the flow chart of the three tools (Vina, QVina 2, and QVina-W). Please refer to the supplementary file for their pseudocode.

To illustrate the theory; consider a search space with (*n*) number of exploring threads, the relative frequency (*Fr*) of the head of thread *i* to pass in proximity to any point of the history of thread *j* at time *t* ≠ 0 is

1 $$Fr(i,j,t)=\frac{{\sum }_{j=0}^{t-1}C({x}_{i},{x}_{j})}{t\ne 0},$$

2 $$C({x}_{i},{x}_{j})=\{\begin{array}{c}1;d({x}_{i},{x}_{j})\le {\rm{R}}\\ 0;otherwise\end{array},$$

where *d(x*_*i*_, *x*_*j*_) is the Euclidian distance between points *x*_*i*_ and *x*_*j*_, and R is a predetermined cutoff. The sum of proximity relative Frequencies (*SoF*) of thread *i* head to pass near to the history of any other thread *j* at time *t*

3 $$So{F}_{it}=\sum _{j\ne i}Fr(i,j,t)$$

is a real number ∈ [0, *n*]. It is important to note that *SoF* can exceed 1.0 (because a thread may pass near to more than one other thread simultaneously). This is particularly common in two cases: 1) near local minima [in pockets], where several threads tend to converge, and 2) progressively towards the end of the search, as all the threads tend to cover the entire search space extensively. The Average *SoF* (*ASoF*) at any time *t* is

4 $$ASo{F}_{t}\,\frac{\sum _{i}^{n}So{F}_{it}}{n}.$$

We expect that tracking *ASoF* over *t* ∈ [0, T] would show a progressing trend.

**Figure 2:**
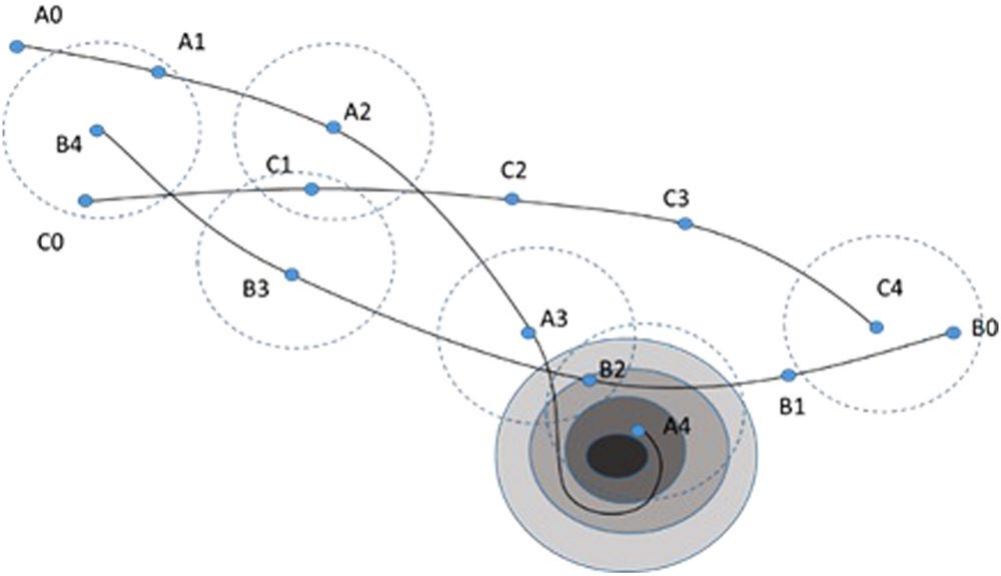
Illustration of progression of threads proximity in 2D. (**A**–**C**) represent the searching threads while 0, 1, 2, 3, and 4 refer to the time point at which the thread is. The dark area at the lower right corner represents a local minimum.

Next, consider the theoretical 2D search space shown in Fig. 2 with three threads exploring it (A, B, and C) and a local minimum in the lower right corner. At *t* = 1, none of the threads are near to the history of any other threads yet, so the *ASoF*_1_ = 0. When *t* = 2, A_2_ was close to the history of C_1_ so A_2_ gets score 1 and the *ASoF*_2_ would equal (1/2 + 0 + 0)/3 = 0.167. When *t* = 3, A_3_ passed by the history of B_2_, and B_3_ passed by C_1_, so *AsoF*_3_ = (1/3 + 1/3 + 0)/3 = 0.222. When *t* = 4, A_4_ was already descending in the well, near to B_2_ again (from the other side), and got a score of 1; B_4_ was near to both A_1_ and C_0_, thus got a score of 2; while C_4_ was close to B_0_, getting another score of 1. Therefore, *ASoF*_4_ = (1/4 + 2/4 + 1/4)/3 = 0.333. Consequently, the progress of the *ASoF* would be (0, 0.167, 0.222, 0.333), which increases with time. Our hypothesis is that the increase in *ASoF* is associated with increased speed and accuracy of the decision making, as we will elaborate using an example later.

### High-quality history points

An essential question here is which points we should keep from the history of all threads. We decided to use the output of last iteration of local optimization (also known as end points or stop points). Typically, these points had undergone up to 300 cycles of essential local optimization then up to other 300 cycles of local optimization (refer to Fig. 1 and supplementary data). Therefore, each of these points is the lowest along the direction of gradient of up to 600 other points. Being the lowest implies being the nearest to the local minima (if any) in this area.

### Data

We tested our theory on the core set of PDBbind 2015, which includes representative 195 protein-ligand complexes^15–17^. We used MGLTools^18,19^ to prepare the ligands through command line, by converting all ligands and receptor files into PDBQT, and enabled all rotatable bonds (backbone phi and psi, amide, and guanidinium). To avoid any possibility of bias, the ligands were then randomized using vina “–randomize_only” parameter, to generate random starting respective poses different from the experimental ligands. Moreover, for every complex, we used the same stochastic search seed for both the small and large search spaces and for all the tested configurations.

We then validated QVina-W (in its final configuration) by virtually screening the 54520 structures from Maybridge library screening collection against the crystal structure of influenza A H1N1 Nucleoprotein (NP) chain A monomer, obtained from protein data bank (PDB ID: 2IQH), comparing the results to those obtained from Vina on the same structure.

### Search space

We had two search space settings: one for searching a certain pocket (referred to as small search space hereinafter) and the other for searching the whole receptor surface without any preference to any pocket (referred to as large search space hereinafter).

The small search space is similar to that defined in Vina, QVina 1 and QVina 2^4,8,9^. For each of the 195 complexes of the test set, the search space is defined as the minimal rectangular parallelepiped, aligned with the coordinate system that includes the docked ligand, plus added 5 Å in the 3D (three dimensions: X, Y, and Z). Additional 5 Å were added randomly to either sides in each dimension, to decentralize the search space over the target pocket. If the search space in any dimension was less than 22.5 Å, it was uniformly increased to this value to ensure the search space allowed the ligand to rotate. For the influenza A NP, we used the T-loop binding site used in Awuni *et al*.^20^.

We defined the large search space (for both the PDBbind core set and Influenza NP), by determining the largest dimension of the ligand, and adding its value to the protein at both directions in each of the three dimensions, following the recommendation that the ligand should be allowed to rotate in the search space^19^. We did not take any other measures to decentralize the search space because it is already centered on the protein center of geometry, while the target pocket is somewhere on the protein surface.

### Procedure overview

After preparing the benchmarking dataset, we profiled the performance of QuickVina on the dataset using different configurations of internal tool parameters on small search space, in order to select the best candidate configuration. Afterwards, we projected that configuration to large search space, where we applied the inter-process communication, and profiled its performance. Then we kept increasing its maximum number of steps, until we reached four folds of the number of steps the original Vina undergoes. We will describe the procedure steps in detail in the next sections.

### Development of QuickVina-W

#### Profiling different QVina 2 on small search space

Vina has a parameter called “exhaustiveness” that controls how comprehensive its search is. The more the exhaustiveness, the less the probability a good result is missed. Throughout the profiling, we kept the exhaustiveness value equal to the number of CPUs used. We changed the code of QVina 2 to test different configurations. These configurations include maximum number of checks (P) mentioned earlier and buffer size (Q). For our study, we tested different combinations of configurations {(P, Q)}, as well as exhaustiveness level (E). Configurations are {(P, Q)| P ∈ {0.5 N, N, 2 N, 4 N, 6 N, 8 N} AND Q ∈ {N, 2 N, 5 N, 10 N, 20 N, 40 N} AND P ≤ Q}. Combinations are in the form {((P, Q), E) | E ∈ {8, 16, 32}}. The value N is the number of degrees of freedom (equals six + number of rotatable bonds). For example, suppose that QVina 2 is run with configuration (P = 4 N, Q = 5 N) and exhaustiveness (E = 16). When the ligand has 4 bonds, there will be 4 + 6 = 10 degrees of freedom (N), so the maximum number of checks would be the nearest 40 (4 N) points among the latest 50 (5 N), for each of the 16 threads exploring the search space.

After small search space profiling, we selected the configuration that showed the best results to project it to the large search space. The selected configuration was P = 4 N, Q = 5 N. We then used exhaustiveness value of 64 in the large search space. Details of the profiling process is available in the supplementary document.

#### Adding the Hybrid Buffer and profiling on large search space

We kept the maximum number of checks and the buffer size to 4 N and 5 N respectively. We modified the QVina 2 code to add a global buffer in addition to the individual buffer for every thread. We refer to the combination of both the global and individual buffers as the **hybrid** buffer, which means for a thread to check whether a potential point is significant or not, it would check the nearest points in the **global** buffer first. If not detected as significant, or if there are not enough near points in the global history to decide, then the thread searches the history of its own **individual** buffer next.

We then profiled the two parameters of the new check: the proximity cut off radius (R) and the maximum number of checks allowed from the global buffer (P_1_ ≪ P). The proximity cut-off is calculated as the Euclidian distance in the three dimensions.

The number of checks *actually performed* from the global buffer is (p_1_ ≤ P_1_). After p_1_ checks are done, the rest of P checks (p_2_) are taken from the thread’s own individual buffer. Please note that p_2_ = P − p_1_ (not p_2_ = P − P_1_). In both steps, the history points to be checked are **ordered** from nearer to farther according to their Euclidian distance to the potential point in all N dimensions. To extend the previous example with selected configuration (P = 4 N, Q = 5 N) and a ligand with four rotatable bonds, the total maximum checks to be done (P) is 4 N (i.e. 40 checks). Now, if the maximum allowed checks from the global buffer P_1_ is N (i.e. 10) and none of them passed the test, then the remaining 30 checks are completed from the individual thread buffer. If there are only 8 points in the history of the global buffer within the cut off R, then the individual checks p_2_ will be 32.

The best configuration we found was that with P_1_ = 1 N, R = 5 Å, P = 4 N, and Q = 5 N as shown in the supplementary document.

#### Increasing the maximum number of steps

Applying the hybrid buffer boosted a leap of speed in QVina 2 without loss of accuracy. We made use of that boosted search speed without compromising the accuracy, and increased the accuracy further, by increasing the maximum number of steps (S) optimization iterations Vina undergoes. S is determined as a function of the ligand number of movable atoms and rotatable bonds. At a first sight, it seems that increasing S would increase the total duration of the search, and would slow the searching speed. However, as we showed earlier, the more steps taken so far (s ≤ S), the higher the probability to pass nearby high-quality history points, and consequently, the faster (and the more accurate) the decision-making will be. That is to say, provided T_0→1000_ is the time taken to do steps S_0→1000_, if we duplicate S, then T_0→2000_ < 2 * T_0→1000_ as we will show in the results.

We elected the configuration with the best results so far (P_1_ = 1 N, R = 5 Å, P = 4 N, and Q = 5 N) and kept increasing the number of steps S, expecting the accuracy to increase, and keeping in mind to preserve the speed faster than – or at least comparable to- that of Vina, until we reached up to 4 folds the number of steps.

#### Paralellizing the preparation stage

To ensure that the new tool is efficient for whole protein surface sampling, we additionally decreased the tool overhead time by adding multithreading to its preprocessing stage, when it prepares the ligand, the receptor, and the scoring grid.

#### Implementation Details

All angles are measured in radians and normalized to the range [−2π, 2π]. Translation distance is measured in Angstrom.

The newly added global history buffer class responsible for the inter-process communication is a single object that implements the singleton design pattern. The buffer size would increase without limits. However, as long as it would hold a relatively small number of points, the size is not an issue. The global buffer is implemented as an octree (octal tree) of history points. The octree root is a cell that spans over the whole search space; and the history points are distributed in the octree according to their spatial distribution in the three dimensions. The choice of the Octree data structure to store the history points from all threads is related to the fact that blind docking is a [spatially-non-focused search]. Therefore, injecting spatial orientation to enforce spatio-temporal integration necessitates choosing a data structure that best performs in relation to the 3D position, which is the octree.

**Figure 3:**
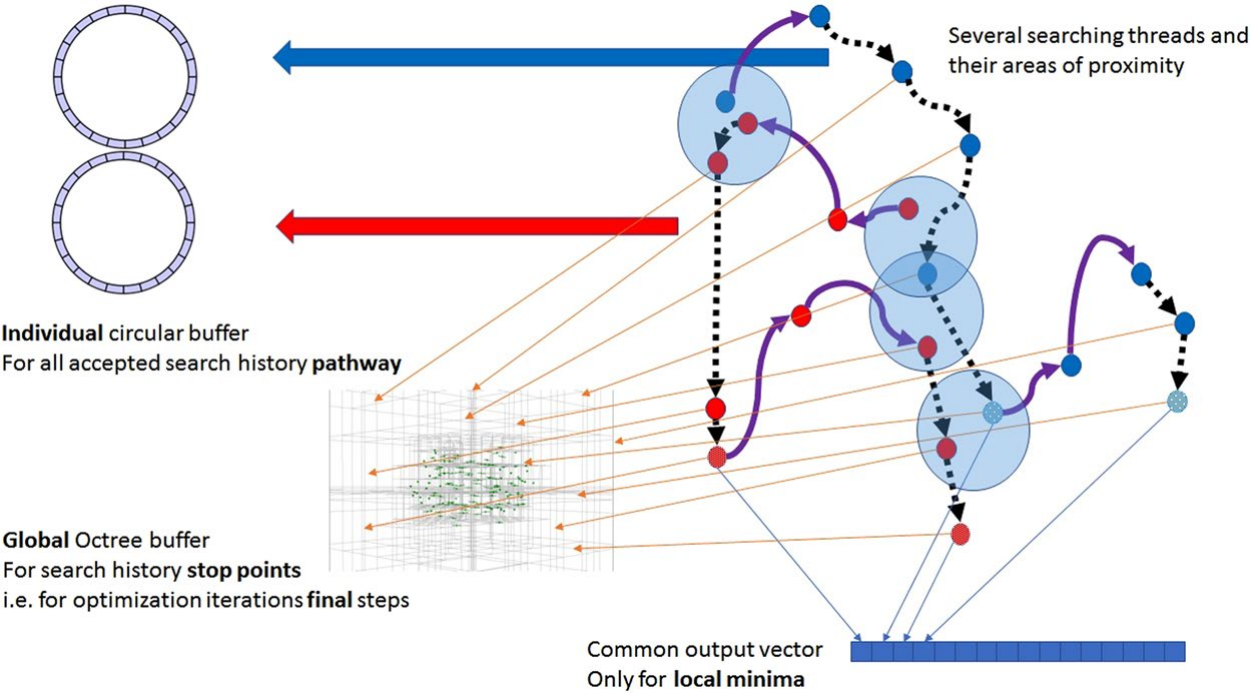
Overview of the whole tool with both types of buffers. Searching threads are shown in violet solid arrows (for Monte Carlo optimization) and black dotted arrows (for BFGS optimization). End (stop) points are shown as small red/blue spheres. Areas where threads come close to each other are shown as light blue spheres. All history points are continuously added to the circular buffer, while end points are stored in the Octree according to their three-dimensional position. Finally, the local minima are added to the results vector.

Figure 3 shows an overview of the whole tool after putting all the parts together. While the searching threads explore the search space, all history points are continuously added to the circular buffer, while end points are stored in the Octree according to their three-dimensional position. The local minima are added to the results vector.

With a fixed proximity cutoff, the tree traversal and processing time increases with increasing the limit of maximum number of contents a node possesses, because that increased limit implies more unnecessary tests. While on the other hand, decreasing the maximum cell (node) content limit in a recursive binary search causes longer processing time, because it implies deeper recursive search overhead. That means one has to balance between the depth and the breadth of the search.

We managed this tradeoff by having two limits, one on the minimum cell size width (W_MIN,_ given an arbitrary value of 0.1 Angstrom) and another one on the maximum number of points a cell can contain (S_MAX,_ given the value 8); and giving W_MIN_ a higher priority over S_MAX_. This way, every leaf node can accept up to S_MAX_. Every time a new point is added to a full (containing S_MAX_ points) leaf node, this node is converted into an internal node and is split into eight leaf children, unless the node is too small to be divided (i.e. unless each cell dimension W_i_ in the 3D is less than W_MIN_). In such a case, the node will not be divided. Instead, the new point will just be added and the node will contain more than the default S_MAX_ capacity. We made this decision because that condition usually occurs around the local minima, where nodes tend to accumulate very close to each other (note that binding pockets are global attractors). In this case, 1) Searching such area will be slow, because it will go so deep (down to 14 levels according to our primary experiments), and the cell width might fall beyond the capacity of C ++ float type precision. 2) Most –if not all– of the adjacent nodes should be considered for checking and consequently there is no need for recursive calls overhead.

When a new potential point is proposed, the currently held points in the tree are filtered according to the Euclidian distance in the first three dimensions **(**i.e. in the spatial distance) to the new point. Then, those which are within the cut off are ordered according to the Euclidian distance (in all dimensions) to the new point from nearer to farther. Points that pass the local optimization step are added to the octree (the history buffer). Lastly, we synchronized reading/writing to the octree using C ++ 09 Shared Mutex.

#### Collecting Results to Analyze

For the PDBbind dataset, we collected the output data as PDBQT files, and compared our results to the experimental data. We used OriginLab to facilitate studying the several dimensions of the data; (i.e. checks, sizes, and exhaustiveness), in order to compare the results from the combination of configurations.

We studied several performance measurements of the docking process, namely Acceleration, Binding Energy, and Root Mean Square Distance (RMSD).

- The acceleration was calculated by dividing the time consumed by Vina by the time of QVina 2 or QVina-W of the studied configuration. The components of time a tool takes are:
  1. The search time (*T*_*S*_), which is the time taken purely to search for probable solutions. Search time acceleration (*a*_*Ts*_) is calculated as
    5 $${a}_{Ts}=\frac{T{s}_{Vina}}{T{s}_{QVina}},$$
    where *Ts*_*Vina*_ and *Ts*_*Qvina*_ refer to the search time of Vina and QVina respectively.
  2. The overhead time (*T*_*H*_), which is the time necessary to load the input files, prepare for the run, and write output files. All the versions before parallelizing the preparation step share almost identical set of values for overhead time, and all versions after parallelization have another almost identical set.
  3. The overall time (*T*_*O*_): All the clock time taken by the tool process run from start to finish, and equals the sum of the previous two times. The overall-time acceleration (*a*_*To*_) is calculated as
  6 $${a}_{To}=\frac{T{o}_{Vina}}{T{o}_{QVina}}$$
  where *To*_*Vina*_ and *To*_*QVina*_ refer to the overall time of Vina and QVina respectively.
- The binding energy was studied by calculating the energy of corresponding models of QVina 2 and QVina-W configuration(s) against that of Vina for the same complex.
- For the RMSD measure, a prediction was considered to be successful if the RMSD of the predicted pose (as calculated in^4,8,9^) with respect to the experimental structure is less than 2 Å. The percentage of complexes with successful RMSD was calculated over 195 total complexes of the PDBbind database.

In addition, we outputted all the history points from all the threads into separate files to allow our retrospective analysis. We counted the number of passes/fails in QVina 2 acceptance check per both the global buffer and the individual buffer, along with the number of checks per every passed test (we needn’t count the failed tests, because we know it is the maximum). We monitored the progression of the ratio between success in global check and success in individual check.

## Results and Discussion

### Search progressions

**Figure 4:**
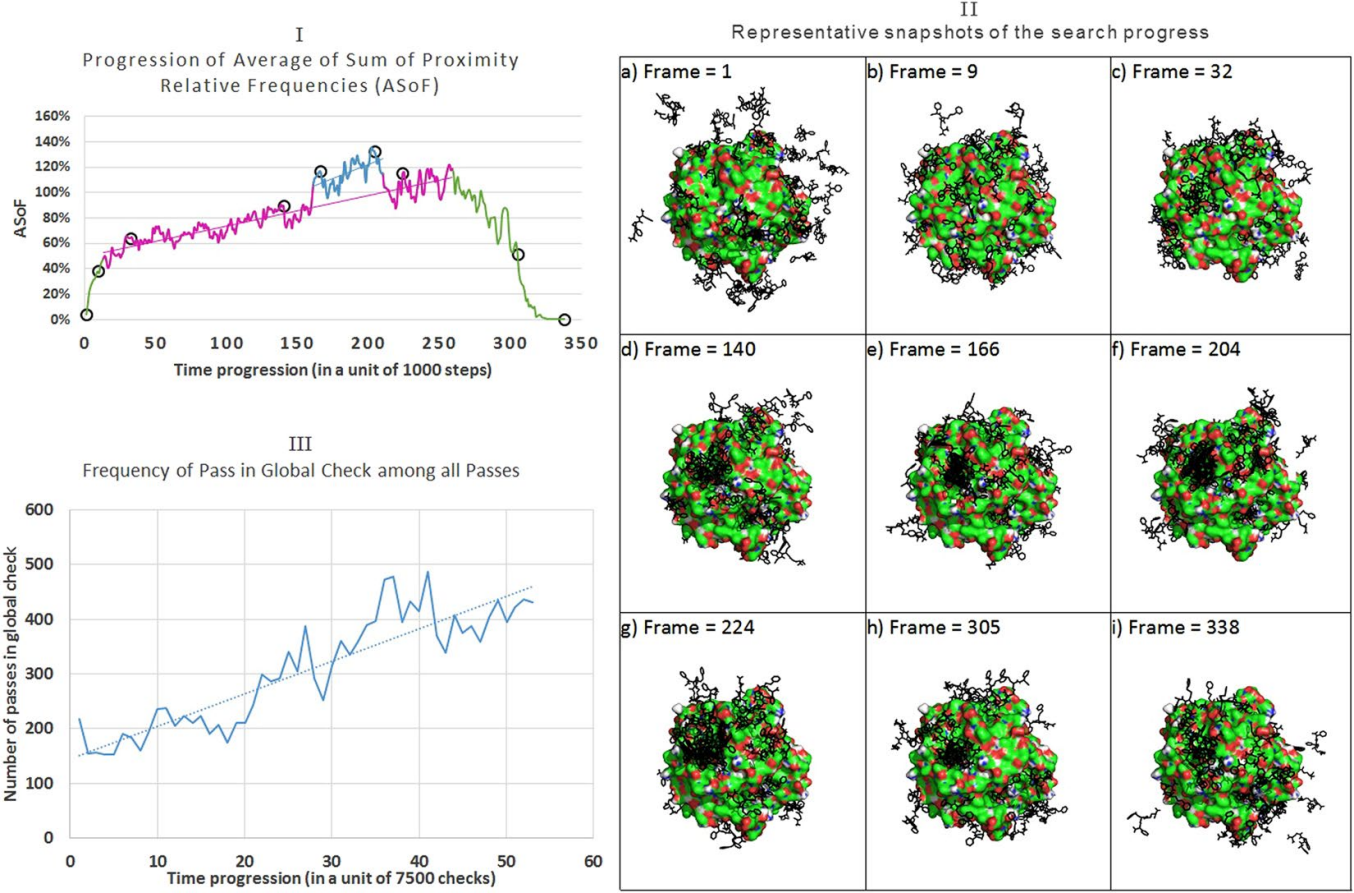
Study of search progress of 10GS. (**I**) Progression of Average of Sum of proximity relative Frequencies (ASoF) of the first 340 time frames. The numbers are averaged over periods of 1000 steps each. The curve shows an initial sharp rising segment and a terminal falling segment (frames 1–12 and 260–340 respectively, both colored in green). Between these segments, a rising trend (frames 13–259, colored in magenta) appears with a slightly shifted small segment (frames 161–210, colored in blue).To investigate and explain the reason of this curve’s features, we mark some representative points with black circles (frames 1, 9, 32, 140, 166, 204, 224, 305, and 338), and show their snapshots in (II). (**II**) Snapshots of the search progress of ligand-protein complex PDB ID 10GS, using 64 threads. The ligands representing the searching threads are in black. (**III**) Progression of Frequency of G test pass among all passes. Every point is the sum of G pass among 2000 passes (either G or I pass) from all threads.

To study the search process, we ran a blind docking search on every complex of the PDBbind Core dataset using 64 threads, and counted the average sum of relative frequencies of any one of the running threads to fall in close proximity of 5 Angstrom to the history “foot print” of any of the other 63 threads (ASoF). In Fig. 4, we study one example complex from our data set (namely PDB ID 10GS). Figure 4(I) shows the progression of that ASoF in the first 340 time frames (each defined as 1000 steps of local/essential-local searches running concurrently), while Fig. 4(II) shows snapshots of the search history at selected points on the graph shown in (I).

Figure 4(I) shows; in the initial loading phase (the corresponding left green segment); that the searching threads start uniformly distributed over the whole search space with a zero ASoF between threads, as seen on Fig. 4(II)a. Then rapidly most of them settle on the surface of the receptor, and consequently their ASoF starts to rise. The second phase (frames 13–259) shows that the search threads explore the whole accessible search space. It shows a steady increase in the ASoF. That goes along with the steady increase in the aggregation of threads, as seen in Fig. 4(II)c–g (roughly proportional to the amount of black dots in clustered structures). One unique feature of this figure was the transient curve-shift seen in frames 161–210 (the blue-colored segment in the middle of the magenta-colored one). By inspecting the search history, we noticed that the reason of this shift is that there was a transient thread (congestion) in one of the cavities. This congestion started, evolved, then resolved. We can see this incident as thread concentration spikes from d (frame 140) to e (frame 166), then the continued increase in f (frame 204) then decrease again in g (frame 224) to a value lower than e but higher than d. The final phase (the right green segment, frames 260–340) is associated with decline in the ASoF. In this phase, the running threads finish one by one and the density of active threads tends to decline, as seen in Fig. 4(II)h. It is noteworthy that the successful threads finish their searches and end earlier, while by the end of the search time, the remaining threads are those that tend to fail in finding suitable solutions and lost their way and tend to explore areas in the search space too far from the receptor surface, as in Fig. 4(II)i.

With increased relative frequency of proximity, we expect an increased rate of passing the QuickVina acceptance check through the global check G. We show the progression of success in that stage among every 7500 passed checks from all concurrent threads, in Fig. 4(III). The figure shows a rising trend. There are two positive expected effects on the increased frequency of G step passes: increased speed and accuracy.

**Figure 5:**
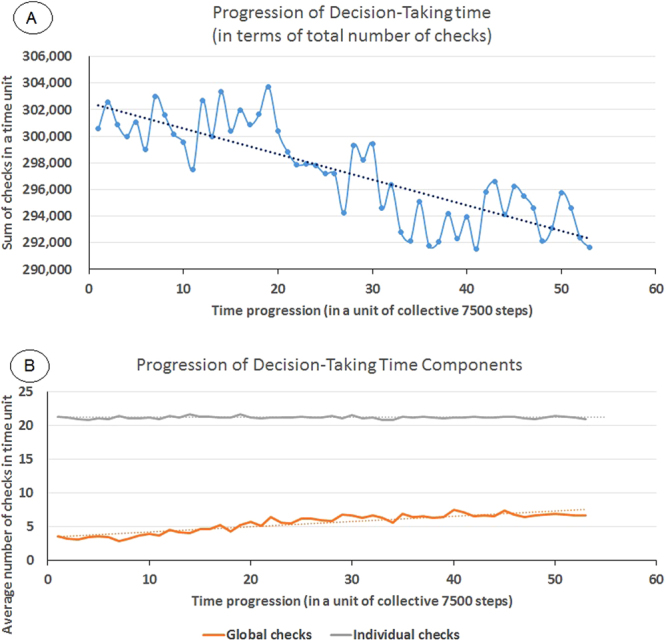
Analysis of decision-making process. (**A**) Progression of decision time, in terms of number of checks taken to pass a potentially significant point, is the sum of checks done in 7500 passed tests. (**B**) Fractional analysis of decision taking time components. The frequency of success in the Global stage increases with time, while the frequency of success in the Individual stage is stationary.

Since the global stage (G) precedes the individual stage (I) and the number of all the performed steps in G stage represents a small portion of the total checks (p_1_ ≤ P_1_ ≪ P), we expect that the more accepted points from the global stage, the less the total checks become, hence less time is required for decision making. Additionally, as more history is accumulated in the global buffer, the search keeps becoming faster towards the end than it was in the beginning. That effect is already found in Fig. 5. In Fig. 5A, we show the progression of sum of checks needed to pass 7500 tests. It is obvious that the number of needed checks per test decreases, which means a significant point is accepted faster to undergo local optimization. To ensure that the declining curve is merely due to the effect of the increased frequency of the G component only, we plotted the different fractions of components participating in the decision taking time in Fig. 5B. The curve shows that the (G) component is increasing while the (I) component is stationary.

**Figure 6:**
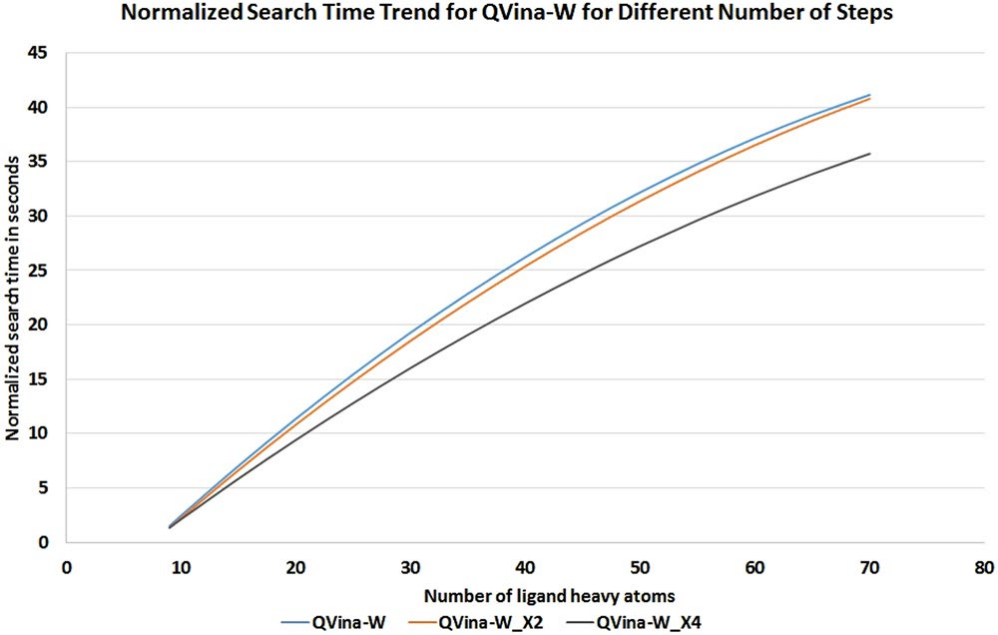
Normalized Search Time Trend for QVina-W for steps X1, X2, and X4. The curve shift from middle to lower curves is much bigger than the shift from the upper to the middle curves, indicating that the search speed keeps increasing as the search progresses, in an increasing rate.

To show the effect of increased search speed on the search time, in Fig. 6 we show the (normalized) search time trend achieved by QVina-W for different numbers of steps S (X1, X2, and X4), in relation to the number of heavy atoms. The figure shows that the (normalized) search time decreases with increasing number of steps S, and that decrease occurs in an increasing rate with increasing S.

**Figure 7:**
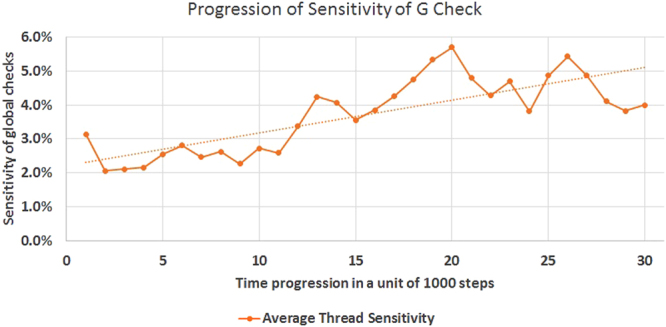
Progression of G check Sensitivity. The sensitivity of the global checks is shown in relation to time in terms of a unit of 1000 steps of the search process.

The second effect of increased ASoF is the decision accuracy in terms of sensitivity. Considering QuickVina acceptance check as ultimate positive condition, we can define the sensitivity of the G phase successes as a rate of acceptance by G step divided by rate of total acceptance (G + I). Again, Fig. 7 shows a rising trend denoting increasing sensitivity.

**Figure 8:**
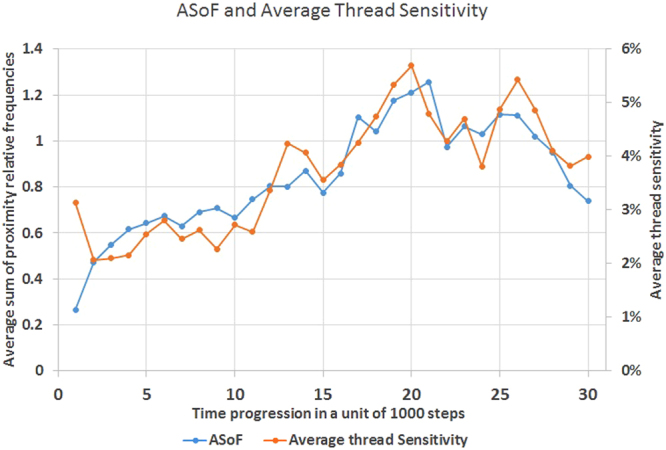
Relation between ASoF and Average thread Sensitivity. A plot of both ASOF and average sensitivity in relation to time in term of a unit of 1000 steps. It shows the definite relation between them with Correlation Coefficient r = 0.862.

Finally, to prove the relation between the ASoF and sensitivity, we plot both the ASoF from Fig. 4(I) and average sensitivity from Fig. 7 together in Fig. 8. The graph shows a definite relation between them with Correlation Coefficient r = 0.862, which proves our theory.

### Increasing maximum steps

After deciding the best configuration of our tool (5Å1N_4N5N as shown in the supplementary file), we tested the effect of increasing the maximum steps on the same best setting after changing the maximum steps to be doubled (×2) and quadrupled (×4).

**Figure 9:**
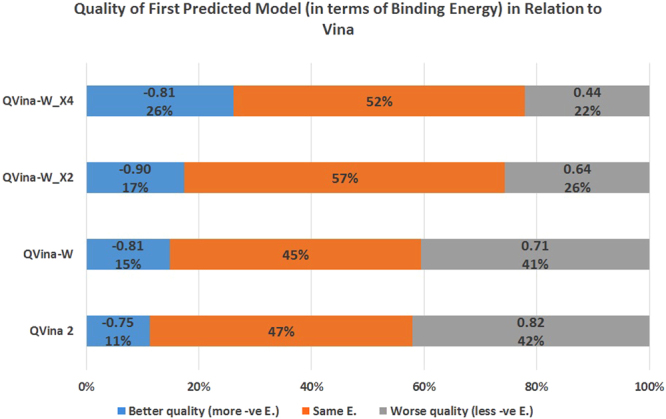
Quality of first predicted model (in terms of binding energy) of different steps of QVina-W and the previously published version of QVina, in relation to Vina. Binding energy of the first predicted model of the previously published version of QVina(QVina 2), and QVina-W with different steps compared to Vina. The decimal numbers on both sides are the average Binding Energy difference.

In term of the binding energy illustrated in Fig. 9, there is an obvious enhancement trend in QVina 2, QVina-W configurations X1, X2, and X4. The later shows 78% of the predictions with binding energies better than or equal to the original Vina 1.1.2, out of which 26% are better with average 0.81 KCal/mol more negative.

**Figure 10:**
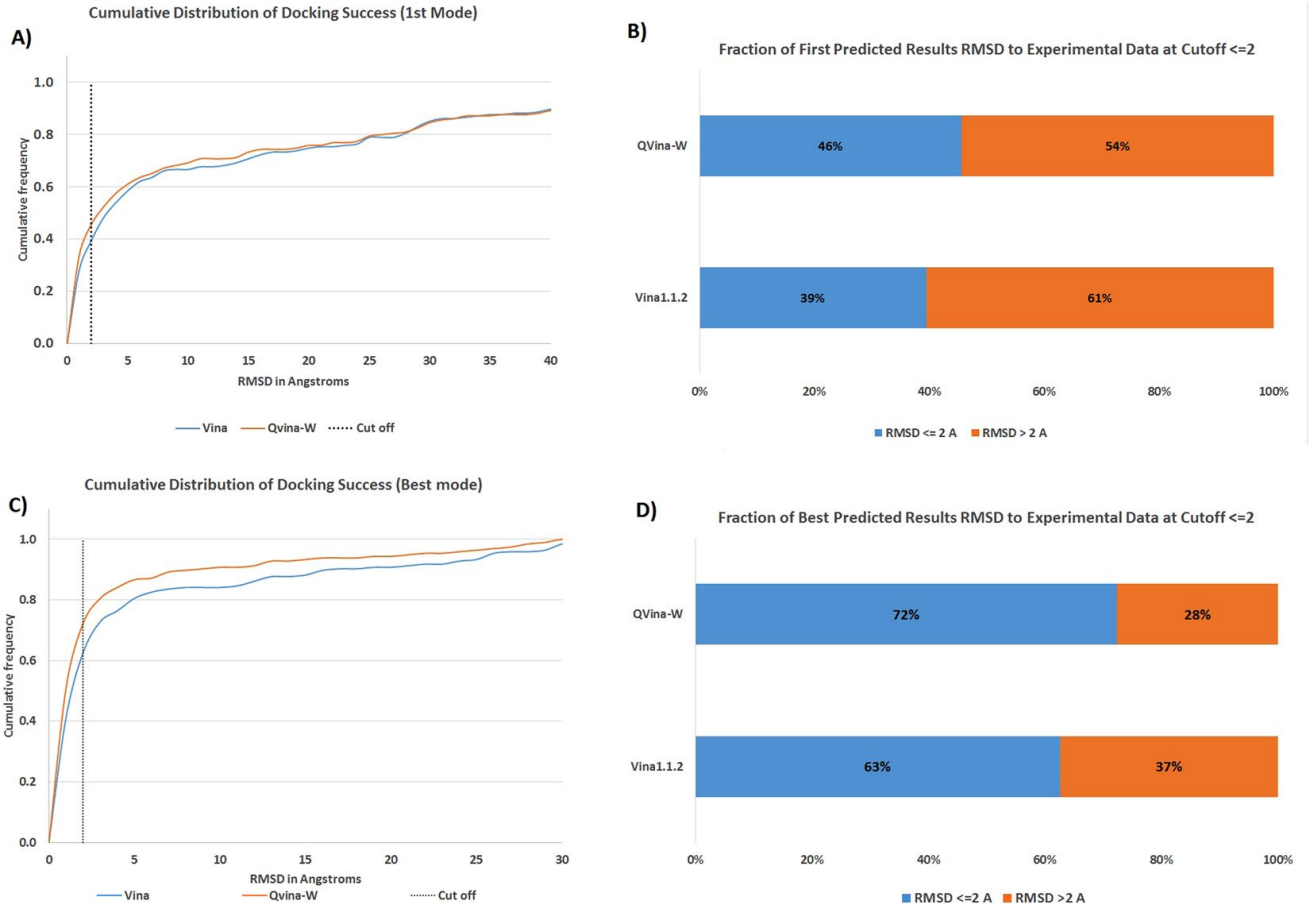
RMSD of Vina, QVina 2, and QVina-W. Relative frequency of successes using RMSD to experimental data for both Vina and QVina-W. (**A**) RMSD distribution of the first mode. (**B**) First mode success at 2.0 A. (**C**) RMSD distribution of the best mode. (**D**) Best mode success at 2.0 A.

Similarly, Fig. 10A shows a clear RMSD success enhancement of QVina-W over the original Vina 1.1.2, with 46% success in the first predicted mode, versus 39% for the Vina predictions. Similarly, Fig. 10B shows that the quadrupled number of steps is by far much better than Vina if we consider any (best) predicted mode (72% for the new QuickVina versus 63% for the original Vina).

It is here worth mentioning that for blind docking experiments over the whole receptors surface, it is useful to consider all predicted modes, not the first one only.

### Overall acceleration after multithreading the preparation overhead

**Figure 11:**
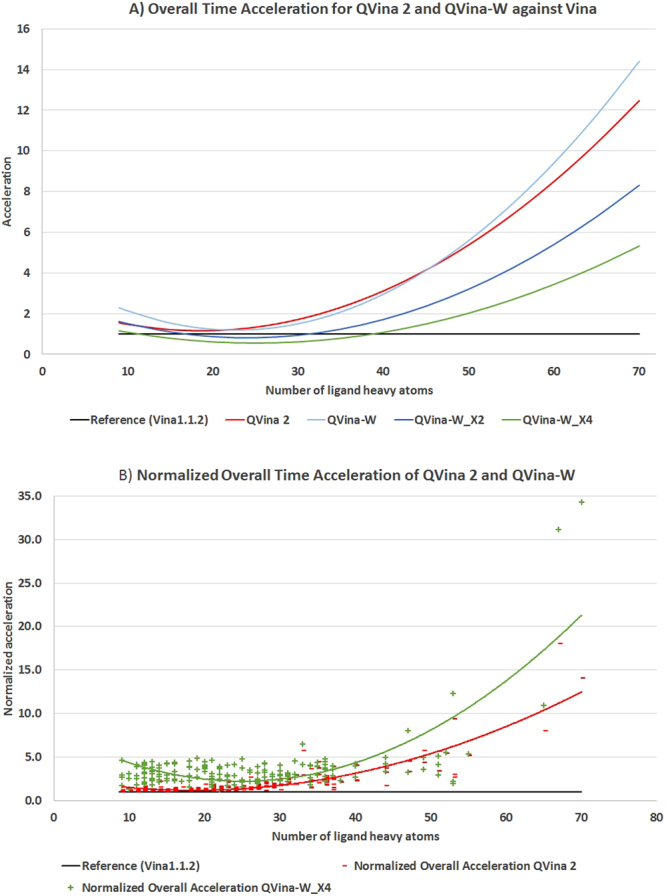
Acceleration of QVina 2 and QVina-W (different steps) against Vina. (**A**) Overall time acceleration for QVina 2 and QVina-W steps X1, X2, X4 against Vina. (**B**) Normalized overall time acceleration for QVina 2 and QVina-W.

As we accelerated the overhead time as well, it is more legitimate to calculate the acceleration based on the overall time rather than the search time only. In Fig. 11A, we show a crude calculation of the overall-time acceleration of previous QVina 2 and QVina-W steps X1, X2, and X4 against Vina. The overall-time acceleration, as calculated in formula (6), shows that the quadrupled QVina-W still has the superiority over Vina when the ligand contains a number ≤11 or ≥39 heavy atoms.

Finally, if we consider that a single run of the QVina-W is effectively equivalent to 4 runs without having to repeat the overhead time, we can normalize the acceleration calculation by means of dividing the QVina time by its base (B ∈ {1, 2, 4}). This way, the calculation would be

7 $$normalized\,{a}_{To}=\frac{T{o}_{Vina}\ast B}{T{o}_{QVina}},$$

where B determines the abovementioned QVina base. The exact normalized acceleration values are displayed in Fig. 11B and summarized in Table 1. They show that the quadrupled configuration is superior to the previous QVina 2 with a maximum normalized acceleration of 34.33 and average 3.60 folds versus respective values of 18.02 and 1.98 for the pervious QVina 2. It is noteworthy that the normalized acceleration average is only less than a factor of 2, because there is a direct relation between the ligand size (number of heavy atoms) and the gained acceleration, but the dataset ligand sizes are not evenly distributed. The histogram of number of heavy atoms, as seen in the supplementary data, is right skewed, with 58.5% of the set having ≤25 heavy atoms.

**Table 1:** Normalized overall time acceleration values (in relation to Vina).

Normalized overall time acceleration	Average	Maximum
QVina 2	1.98	18.02
QVina-W	3.60	34.33

From the results shown above and in the supplementary data, we can conclude that the latest configuration of QuickVina with global buffer explores four folds points in the search space more than AutoDock Vina and previous QVina 2. It obtained better results than Vina, yet in faster time compared to QVina 2. The better results are in terms of both Binding Energy and RMSD. It is faster than Vina in a crude comparison when the ligand heavy atoms are ≤11 or ≥39; and faster than both Vina and QVina 2 in a normalized acceleration where it scored 34.33 fold maximal acceleration and 3.60 folds average acceleration over Vina 1.1.2. The final configuration is “QuickVina with circular individual buffer of size 5 N, maximum checks 4 N and Octree global buffer with cutoff of 5 Angstrom and maximum checks of 1 N, where N is the number of degrees of freedom”.

We are releasing this tool under the name “Quick-Vina-Wide” (QVina-W), which refers to the ability to work in wide search space. It is suitable for blind docking with its proven high accuracy and accelerated speed.

## Conclusion

In this work, we present QVina-W, a new docking tool particularly useful for wide search space, especially for blind docking. QVina-W utilizes the powerful scoring function of AutoDock Vina, the accelerated search of QVina 2, and adds thorough search for wide search space. It is based on the observation that allowing a searching thread to communicate with other nearby threads to make use of their wisdom, would increase the speed and sensitivity of that searching thread. This communication was allowed by means of a global buffer that keeps high quality search history points from all the threads.

In order to prove our theory, we analyzed the search process to trace the Average Sum of Proximity relative Frequencies (ASoF) among all searching threads, along with its effect on the speed and sensitivity of decision taking, as well as the effect on increasing number of search steps on the search speed and accuracy. That proved the direct relation between the length of the search and ASoF, which is reflected on the search speed and accuracy, and that in turn implies higher probability for better results. QVina-W makes use of the acceleration and explores four folds the number of points that Vina used to explore in a more efficient way. We also multithreaded the preparation overhead, which adds more to the overall time acceleration.

QVina-W proved to be faster than QVina 2 (with average and maximum normalized overall time accelerations of 3.60 and 34.33 folds in relation to Vina versus 1.98 and 18.02 respectively), yet better than Vina in terms of Binding Energy (78% of predictions with binding energy better than or equal to Vina) and RMSD (with success rate of 72% by QVina-W versus 63% by Vina).

### Future work

Our plan to extend this work includes implementing genetic algorithm between nearby points to maximize the benefit of shared wisdom of threads, in addition to making a self-fine-tuning tool for QuickVina, to adjust its parameters according to the installation environment.

### Availability of data and materials

The tool is available from this [http://www.qvina.org]

- Operating system(s): cross platform
- Programming language: C++
- Other requirements: BOOST 1.60
- License: Apache License (Version 2.0)

The dataset supporting the conclusions of this article is available in the PDBbind database repository, [http://www.pdbbind.org.cn].

## Electronic supplementary material

Supplementary Document

Search Progress sample
