# Supplementary material for: Protein-Ligand Blind Docking Using QuickVina-W With Inter-Process Spatio-Temporal Integration
Source: Sci Rep. 2017 Nov 13;7:15451. doi: 10.1038/s41598-017-15571-7 (PMC5684369; doi:10.1038/s41598-017-15571-7)
Supplement: Supplementary file 1 — Supplementary Document [file 41598_2017_15571_MOESM1_ESM.pdf]

Protein-Ligand Blind Docking Using QuickVina-W With Inter-Process Spatio-Temporal Integration

Supplementary Document

Nafisa M. HASSAN ¶[1], Amr A. ALHOSSARY ¶[2], Yuguang MU*[1] and Chee-Keong KWOH*[2]

[1] School of Biological Sciences, Nanyang Technological University, Singapore
[2] School of Computer Science and Engineering, Nanyang Technological University, Singapore

¶ These two authors contributed equally as first author.
* Corresponding author
Email: asckkwoh@ntu.edu.sg, ygmu@ntu.edu.sg

Supplementary Introduction

QuickVina (and subsequently QuickVina 2) are based on decreasing the computational load by preventing the most time-consuming step of the search process (i.e. from refinement by the local search) from being executed, when the new potential point is expected to sink in the same current/last energy basin into the last local minimum. A local minimum -by definition- is a stationary point (where derivatives are zero in respect to all design variables) in the continuous scoring (energy) function. Therefore, standing in a transition zone on the rim of (or falling into) a new basin is indicated by having the first order derivatives of the scoring function with respect to all the design variables to be zero or with opposite signs in relation to all the corresponding variables of at least one of the **[most recently]** visited points. Please notice the **[progressive]** nature of both the Monte Carlo and BFGS methods of stochastic global and deterministic local optimizations utilized in Vina.

Supplementary Figure 1is a visual illustration of a 1D objective function with its derivative in several points. Section I to the left is reproduced from our previous publication (Alhossary et Al. 2015). Section II to the right is a global zoomed out perspective on the function. In Supplementary Figure 1(I), supposing that the database contains point D (maybe among others), and point C is the new potential point. Point C is accepted, because point D has an opposite derivative sign of the recent history point C. Using the same principle, point 1 from Supplementary Figure 1(II) will not be accepted if the database contains point 3 only, because they both would be optimized to point 12, however, point 1 would be accepted if the database contains point 2.

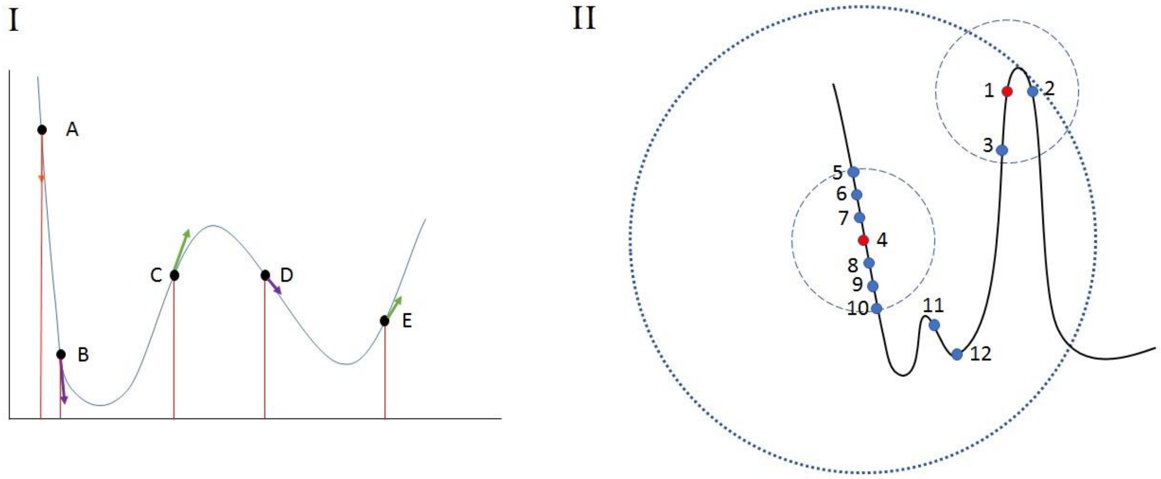

Supplementary Figure 1 One dimensional function illustration of QuickVina principle

Section I (left) shows 1D function with some points along with their function derivative in respect to the design variable. Section 2 (right) shows two points with their neighbours: point 1 may be accepted while point 4 is never accepted.

On the other hand, if only point B exists in the database, and point A is the new point, it is not accepted, because it has the same derivative sign as B and the slope is in the same direction of the sign. Hence, it is not guaranteed to have any stationary points in between them (this may lead to false negatives). It is noteworthy that the derivative value zero is considered a third distinct value and it is not considered neither positive nor negative.

QuickVina 2 introduces a more robust check with much lower false negative rate by considering the first-order-consistency-check. For example, suppose the database contains only point B and point D is the new potential one. Although B and D have the same sign, point D is still accepted, because B has a lower value of the objective function than D; which guarantees the presence of two stationary points in between in this variable direction. The same holds for points C and E in the opposite direction.

This is mathematically formulated as:

A point (i) is accepted if there exists a point (j) of the nearest [4N] points with

$$sign\left(\frac{\delta f}{\delta x_n}(i)\right).sign\left(\frac{\delta f}{\delta x_n}(j)\right) \leq 0,$$

or with

$$sign\left(\frac{\delta f}{\delta x_n}(i)\right).sign(\{f(i) - f(j)\}.\{i - j\}) \leq 0,$$

where $\frac{\delta f}{\delta x_n}(i)$ is the partial derivative of (f) in relation to variable (x_n) at point (i).

It is noteworthy that rejecting a point with confidence requires the beforehand availability of plenty enough history points in the database. For example, to reject point 4 from Supplementary Figure 1(I) with enough confidence, we need to have all near points 5-10 with the same derivative sign to avoid the false negative which could happen if the database contains only point 11. The robust first-order-consistency-aware check of QuickVina 2 abates such false negatives, and to minimize (hopefully eliminate) them, when there are not *plenty* enough *near* history points to decide to reject a potential point with enough confidence, the test defaults to accepting it.

On the other hand, there is a tradeoff between increasing number of checks to increase confidence and decreasing gained speed. Also, if we don't confine the checks to a small enough distance cutoff, the test may become useless; because it would test against irrelevant points (e.g. from another pocket). For instance, if we consider a very wide cutoff (the one with finer dashes), point 4 from Supplementary Figure 1(II) would be accepted, only because of the presence of point 2, which is too far to be informatively relevant.

Supplementary Methodology

Data

Supplementary Figure 2 shows the frequency of number of heavy atoms for the PDBbind dataset we used. Obviously, 58.5% of the sample has less than or equal to 25 heavy atoms.

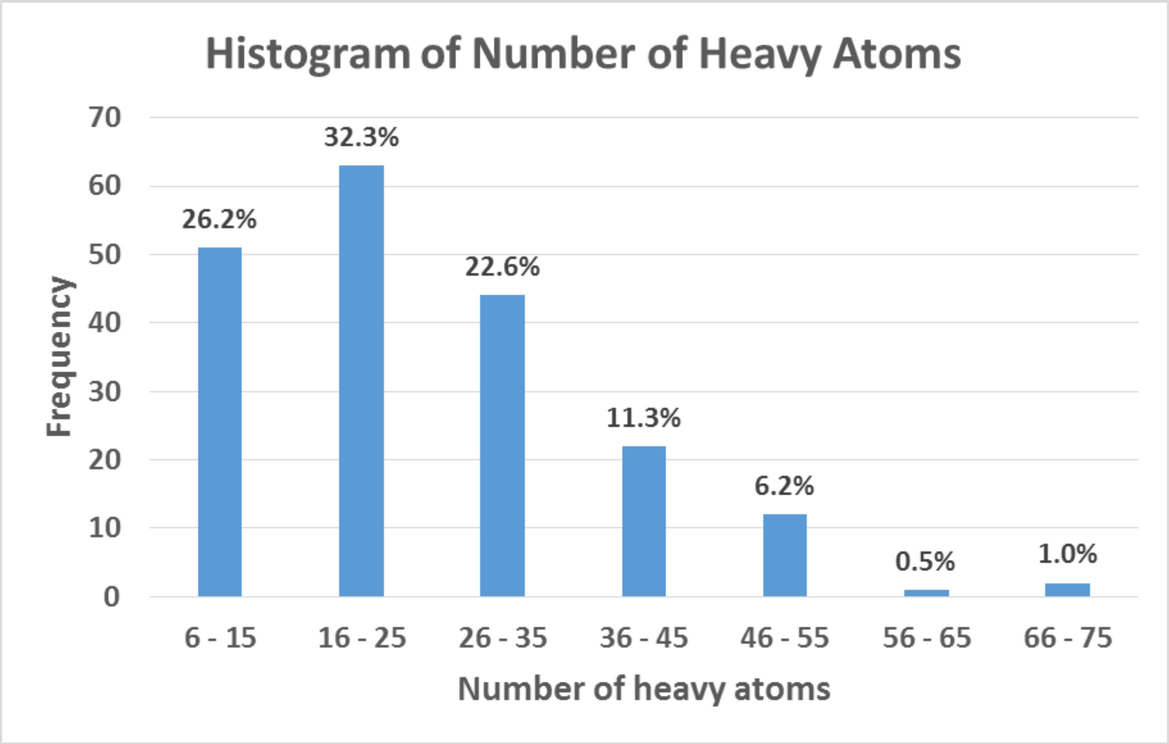

Supplementary Figure 2 Frequency of heavy atoms in PDBBind core dataset.

Optimization in Vina, QuickVina 2, and QuickVina-W

Listing 1: Optimization in Vina

```
Function Search(initial point)
Input:   An initial point to seed the seach
Output:  Last point in the stochastic optimization search
Effect:  Adding some solutions to output vector

FOR STEP = 1 TO Max_Steps
|   CALL global optimization on point X
|   |
|   |
|   |
|   |
|   CALL BFGS for Essential Local Optimization
|   |
|   IF X2 is accepted by metropolis check
|   |   |
|   |   |
|   |   |
|   |   |
|   |   CALL BFGS for Essential Local Optimization
|   |   |
|   |   |
|   |   If X3 is better than previous point
|   |   |   ADD X3 to output
|   |   END IF
|   END IF
END FOR
```

Listing 2: Optimization in QuickVina 2

```
Function Search(initial point)
Input:   An initial point to seed the seach
Output:  Last point in the stochastic optimization search
Effect:  1) Adding some solutions to output vector
         2) Adding all accepted pathway to individual
         buffer

FOR STEP = 1 TO Max_Steps
|   CALL global optimization on point X
|   |
|   IF X1 NOT interesting according to I check
|   |   CONTINUE
|   END IF
|   CALL BFGS for Essential Local Optimization
|   ADD path X1-X2 to INDIVIDUAL History
|   IF X2 is accepted by metropolis check
|   |   |
|   |   IF X2 NOT interesting according to I Check
|   |   |   CONTINUE
|   |   END IF
|   |   CALL BFGS for Essential Local Optimization
|   |   ADD path X2-X3 to N History
|   |   |
|   |   If X3 is better than previous point
|   |   |   ADD X3 to output
|   |   END IF
|   END IF
END FOR
```

Listing 3: Optimization in QuickVina-W

```
Function Search(initial point)
Input:   An initial point to seed the seach
Output:  Last point in the stochastic optimization search
Effect:  1) Adding some solutions to output vector
         2) Adding all accepted pathway to individual buffer
         3) Adding last local optimization point to global
         buffer
FOR STEP = 1 TO Max_Steps
|   CALL global optimization on point X
|   IF X1 NOT interesting according to G check
|   AND X1 NOT interesting according to I check
|   |   CONTINUE
|   END IF
|   CALL BFGS for Essential Local Optimization
|   ADD path X1-X2 to INDIVIDUAL History
|   IF X2 is accepted by metropolis check
|   |   IF X2 NOT interesting according. to G Check
|   |   AND X2 NOT interesting according to I Check
|   |   |   CONTINUE
|   |   END IF
|   |   CALL BFGS for Essential Local Optimization
|   |   ADD path X2-X3 to N History
|   |   ADD X3 to G History
|   |   If X3 is better than previous point
|   |   |   ADD X3 to output
|   |   END IF
|   END IF
END FOR
```

Supplementary Figure 3 Pseudocode of Vina, QVina 2, and QVina-W

State Machine of QVina-W

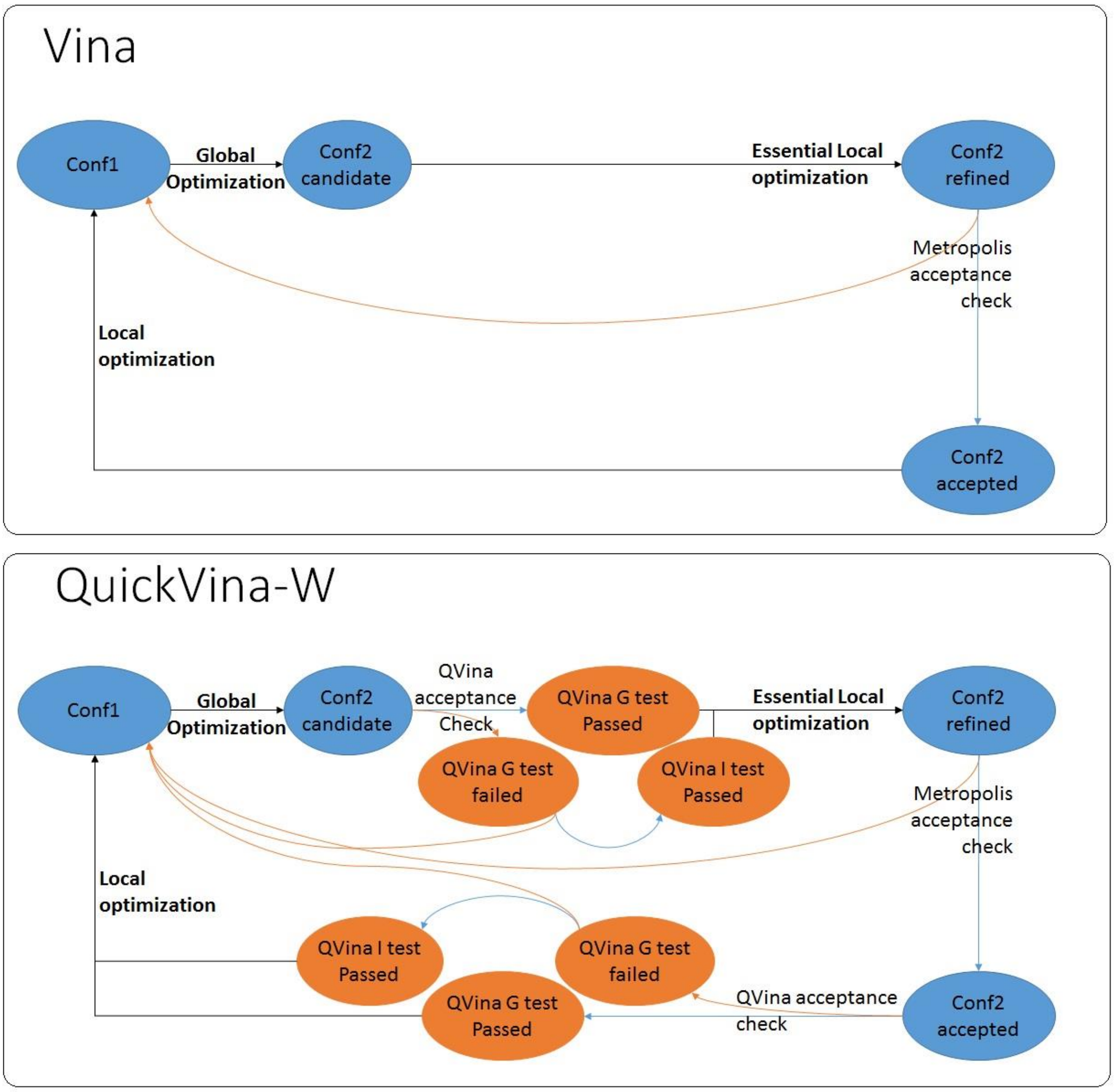

Supplementary Figure 4 State Machine of Vina and QVina-W

State machine of Vina (above), and QVina-W (below). The cycle starts with Conf1. Blue connectors represent passed tests, red connectors represent failed tests, and black lines represent compulsory optimization pathways.

Supplementary Results

Profiling different configurations of QVina 2 on small search space

As the first step in our methodology, we profiled QuickVina 2 on small search space using different configurations. These configurations include maximum number of checks (P) and buffer size (Q). We tested different combinations of configurations {(P, Q)}, as well as exhaustiveness level (E). Configurations are {(P, Q) | P ∈ {0.5N, N, 2N, 4N, 6N, 8N} AND Q ∈ {N, 2N, 5N, 10N, 20N, 40N} AND P ≤ Q}. Combinations are in the form {((P, Q), E)| E ∈ {8, 16, 32}}. The value N is the number of degrees of freedom (equals six + number of rotatable bonds). In Supplementary Figure 5, we show the success rate of different settings using exhaustiveness 16. (Since, Q >= P, we had to fill the missing entries in the graph, by duplicating the last entries in each line where P > Q, in order to get clearer figures). Our experiment showed that the maximum checks of 2N is the junction between high-slope and plateau sections of the surface, and 4N is a safe margin, after which no significant improvement occurs despite of changing the maximum size and exhaustiveness. Consequently, we used 4N as the maximum check, and investigated more to determine the size to be combined with 4N, in order to give the best results in terms of acceleration, binding energy and RMSD. Our profiling showed that the settings of maximum checks (P) of 4N and a maximum size (Q) of 5N was the configuration with the best results obtained as will be discussed later.

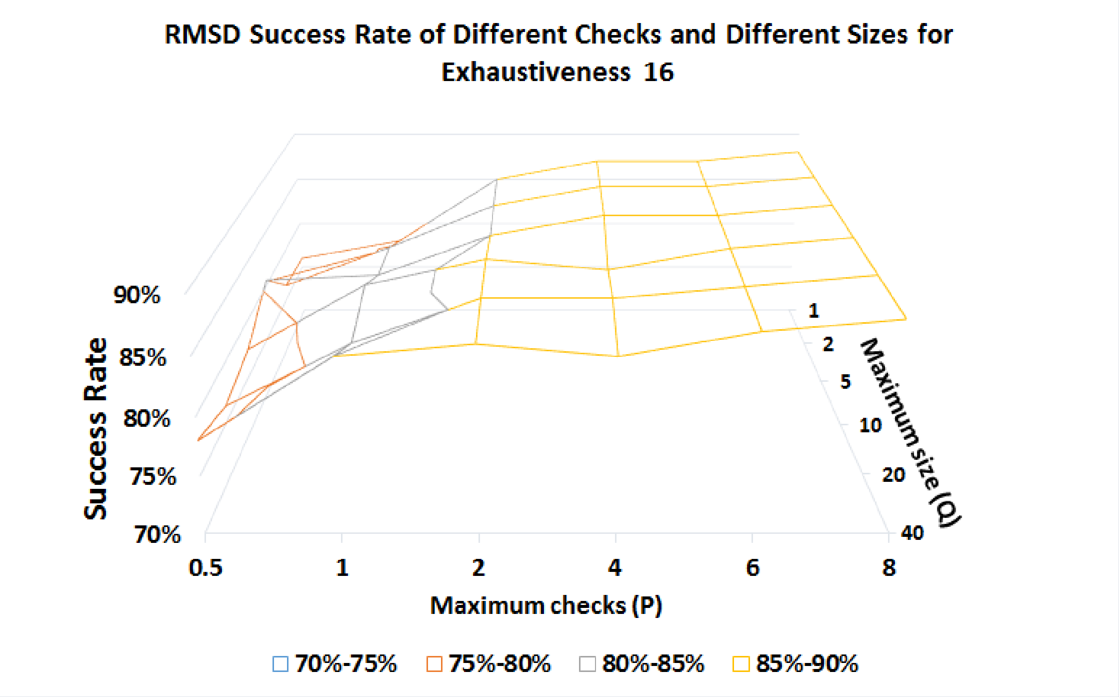

Supplementary Figure 5 RMSD success rate of different checks and different sizes for exhaustiveness 16

In order to find the best Q, we show in Supplementary Figure 6 the search time acceleration of 4N checks along different sizes as calculated in formula (5) in the main text. Buffer size of 5N shows the highest acceleration results among all sizes and shows that with increasing the buffer size, the speed decreases, because it implies more time for sorting and selecting more history points.

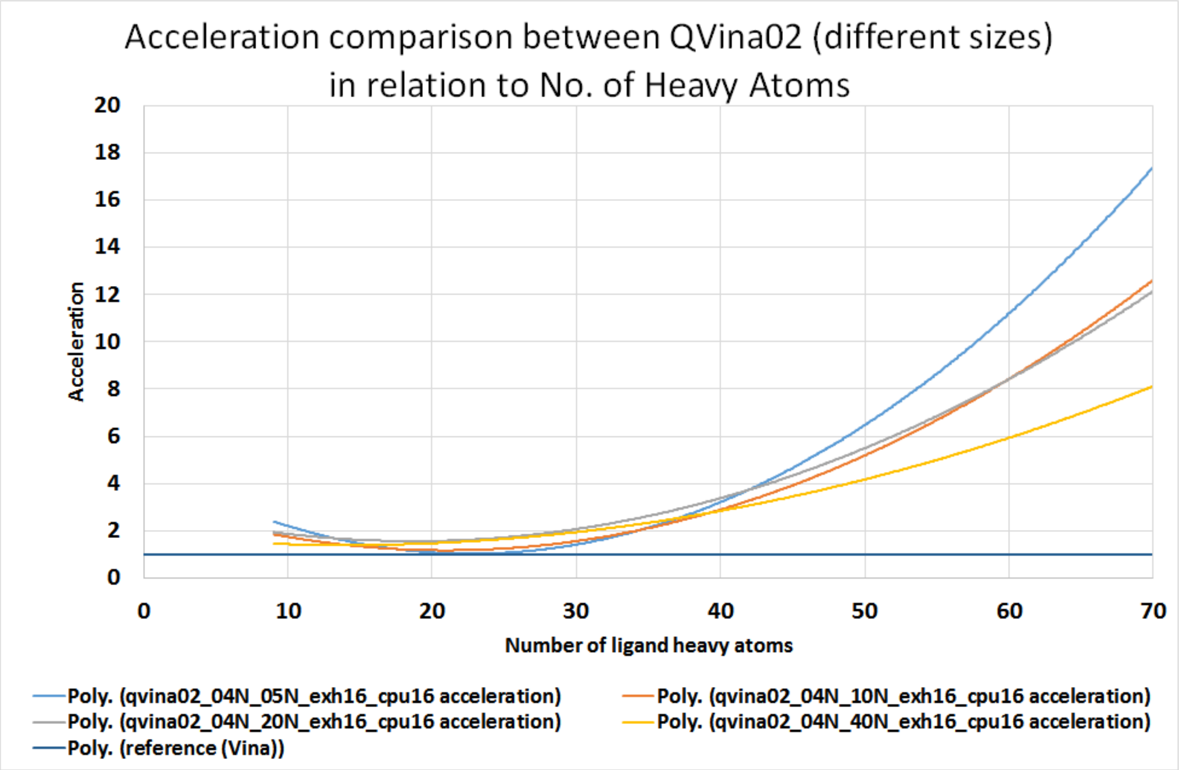

Supplementary Figure 6 Acceleration of QuickVina 2 for checks of 4N with different sizes

Acceleration of QuickVina 2 for checks of 4N along different sizes, in relation to the number of heavy atoms using exhaustiveness 16 (Q ∈{5, 10, 20, 40})

Although the setting of 4N_5N did not show the best results in terms of binding energy as seen in Supplementary Figure 7, the average of "worse" binding energy differences was the lowest among all of them except for the 4N_40N, (which is by far the slowest). In addition, the RMSD of size of 5N was the best among other sizes with checks of 4N as shown in Supplementary Figure 8. Consequently, the good acceleration, the relatively low binding energy difference average, and the RMSD of 4N_5N encouraged us to build upon this configuration for the next steps. The notations of the legend for the mentioned figures are as follows: Vina is the original Vina 1.1.2 results; Qvina02_P_Q refers to the previous QuickVina 2 before adding the global buffer with maximum checks (P), and maximum sizes (Q), the exhaustiveness (16) and number of used CPU (16) are coded as well.

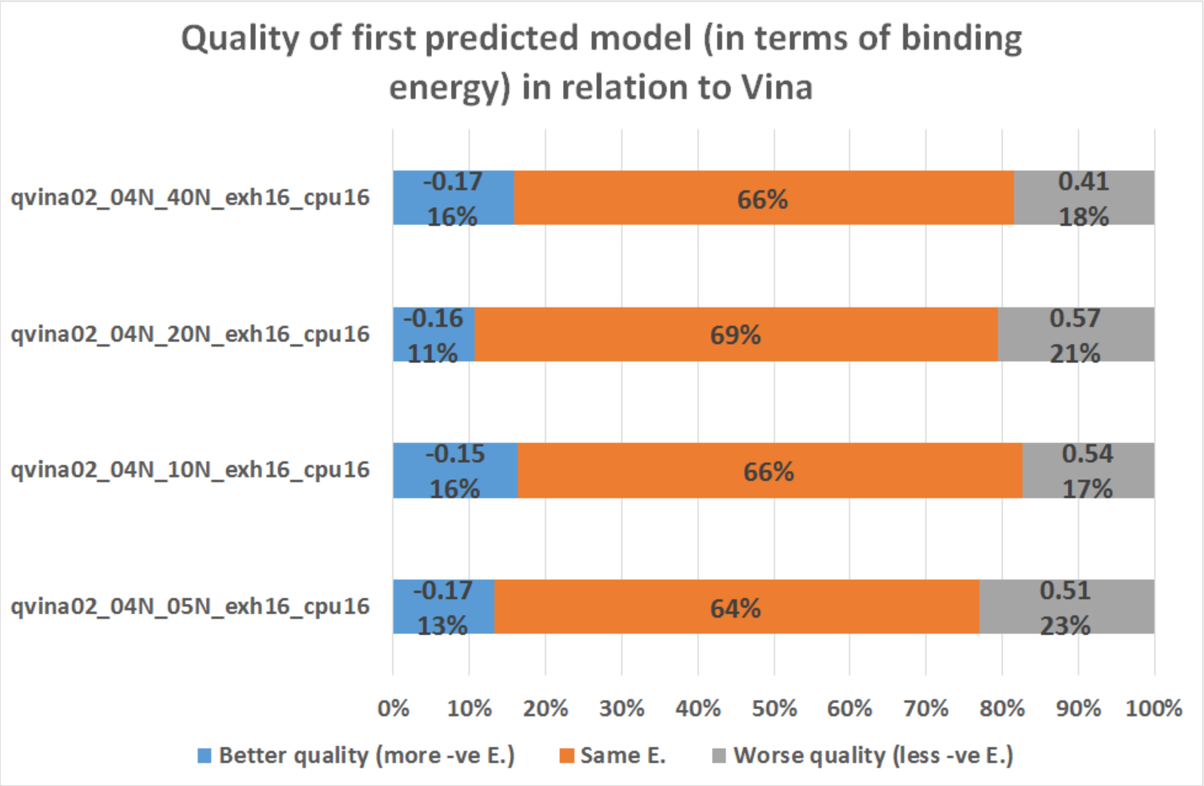

Supplementary Figure 7 Binding Energy of checks of 4N (small search space)

Binding Energy of checks of 4N along different sizes of QuickVina 2 for small search space using exhaustiveness 16.

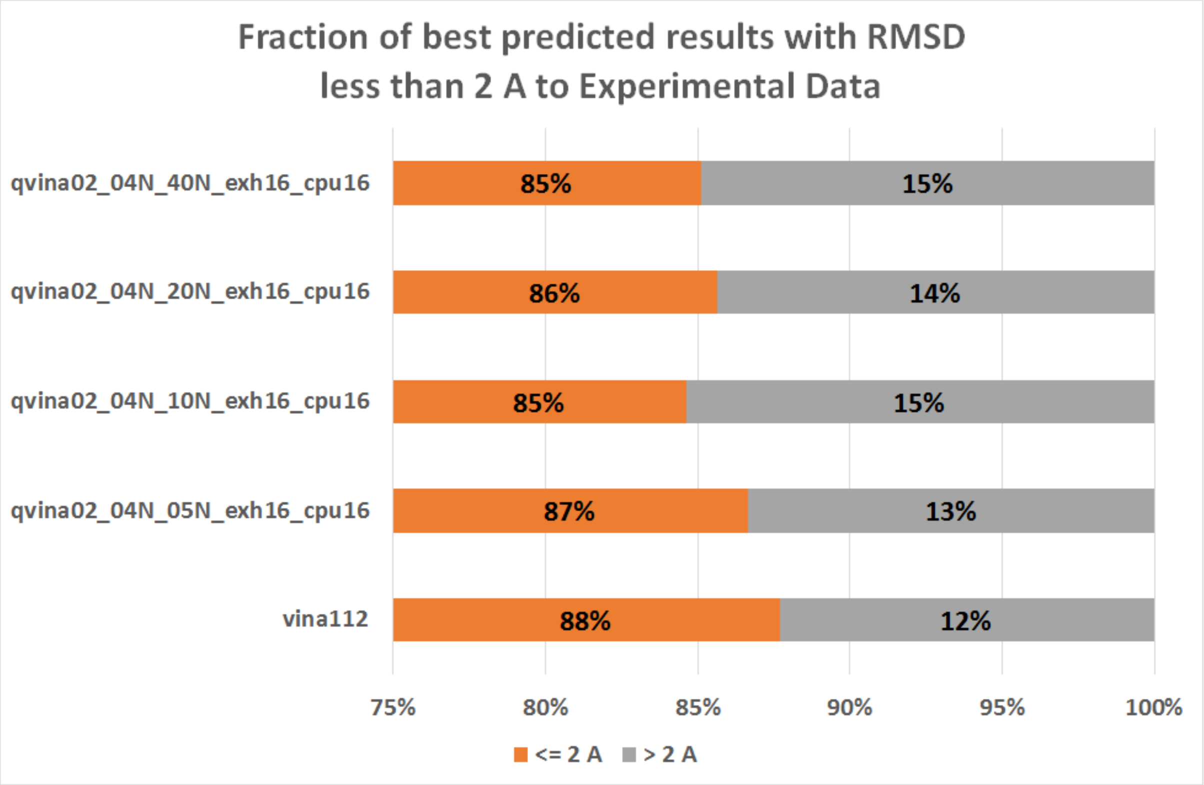

Supplementary Figure 8 RMSD of any (best) predicted results to experimental data in small search space

RMSD of any predicted result to experimental data for QuickVina 2 of checks 4N and different sizes for exhaustiveness 16.

Success is determined as distance <=2 Angstroms, and failure as distance>= 2 Angstroms.

Large search space with hybrid buffer (individual circular + global octree)

All the coming figures hereafter are studied on exhaustiveness of 64 and 64 CPUs as well. Supplementary Figure 9 shows the acceleration of different configurations of QVina02 after adding the octree. The notations of the legend are as follows: QVina02-octree_RP$_1$_PQ represents the updated QuickVina 2 after adding the global buffer, P maximum checks, and Q maximum sizes plus R and P$_1$ referring to the cutoff radius in Angstroms, and the portion of maximum checks allowed from the global buffer respectively.

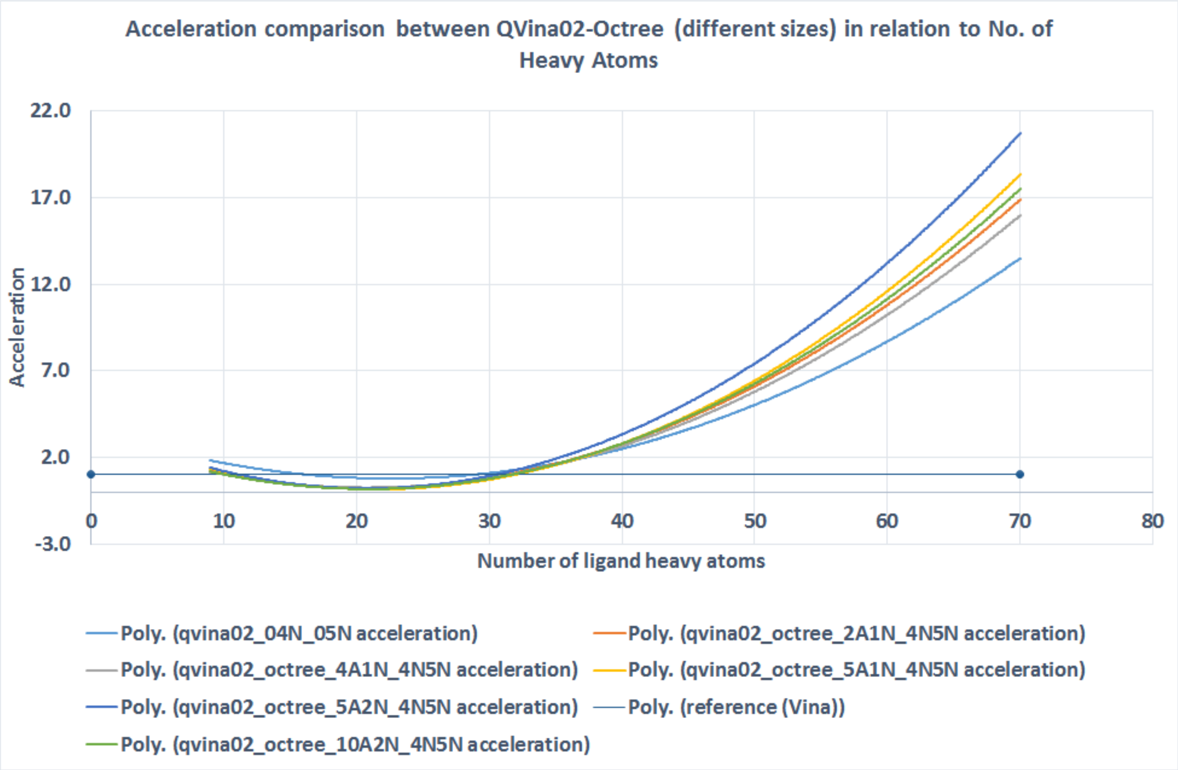

Supplementary Figure 9 Acceleration comparison between different configurations of QVina-W in relation to No. of Heavy Atoms

Acceleration between different configurations of QVina-W in relation to the number of heavy atoms. Qvina02_4N5N is old QuickVina 2 before adding global buffer. The notation QVina02-octree_RP1_PQ refers to: qvina02_octree: QuickVina after

adding the global buffer, R: the cutoff radius in Angstroms, P1: the number of allowed checks from the global buffer, P: the total checks from both buffers, and Q: the size of the individual buffer.

The accelerations of all QVina-W configurations are better than QuickVina 2 accelerations as shown in Supplementary Figure 9. As one must expect, we are mainly concerned with the quality of the prediction rather than the speed. Therefore, although the radius of 5Å and 2N as maximum checks of the global buffer seems the fastest, we chose the configuration of a cutoff radius of 5Å and 1N of the global buffer because it showed better results regarding both the binding energy and RMSD.

The quality of prediction is shown in the next two figures. In terms of binding energy, Supplementary Figure 10 illustrates the percentage of the first models that show better quality (more negative energy), the same binding energy, and worse binding energy, in comparison to the original Vina. In spite of the good results (in terms of binding energy) obtained from the configuration of 10Å, and 2N checks (33% only worse binding energy), it did not show similar good ranking regarding the RMSD calculations as illustrated in Supplementary Figure 11. The configurations 2A1N, 5A2N, and 5A1N share next lowest value (36% worse binding energy). Similarly, configuration of 5Å2N was the best regarding the acceleration, yet the configuration of 5Å1N is better from the binding energy view as it showed the highest portion of better quality (19%), as opposed to 14% and 16% for 5Å2N and 2A1N respectively. On the other side, Supplementary Figure 11 shows that it (5Å1N) scored the best RMSD among all other settings, with success of 63% similar to the original Vina. Consequently, we finalized the settings to be 5Å for the radius, 1N allowed checks from the global buffer, and 4N checks, and 5N size respectively.

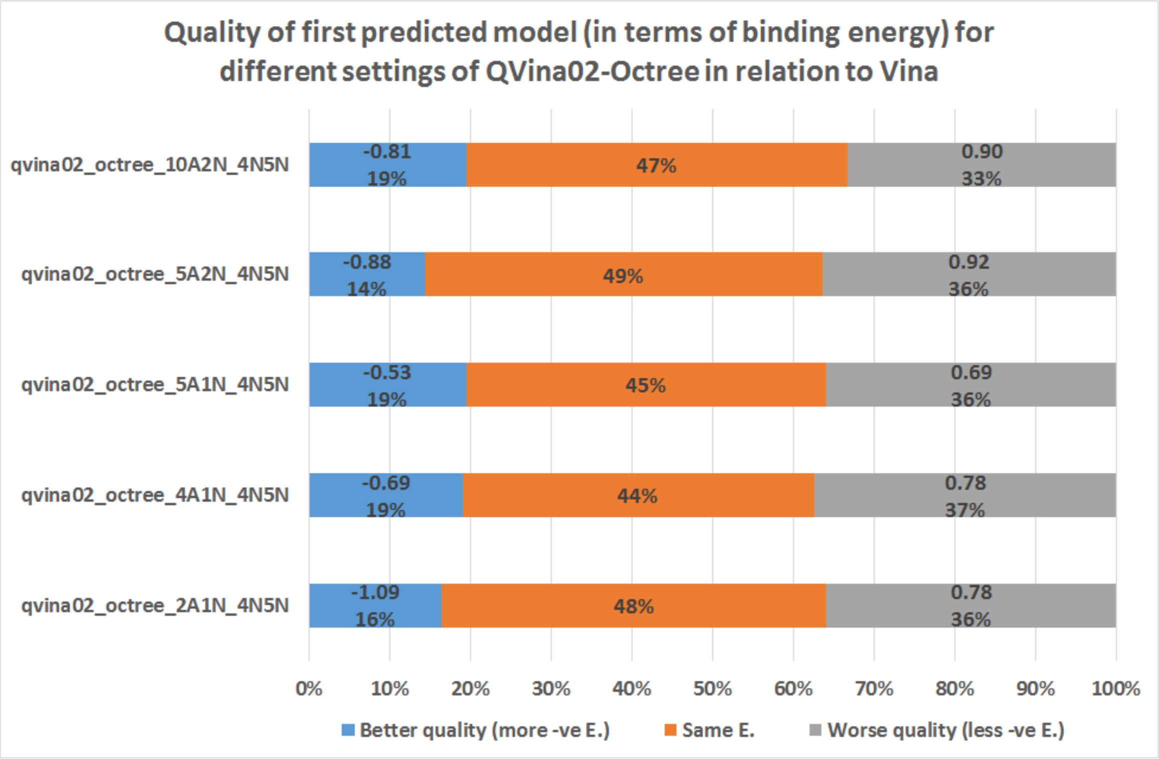

Supplementary Figure 10 Quality of first predicted model (in terms of binding energy) for different configurations of QVina-W in relation to Vina

Binding energy of the first predicted model for different settings of qvina with octree (QVina-W). Averages of binding energy differences in both better and worse quality are given above the percentage.

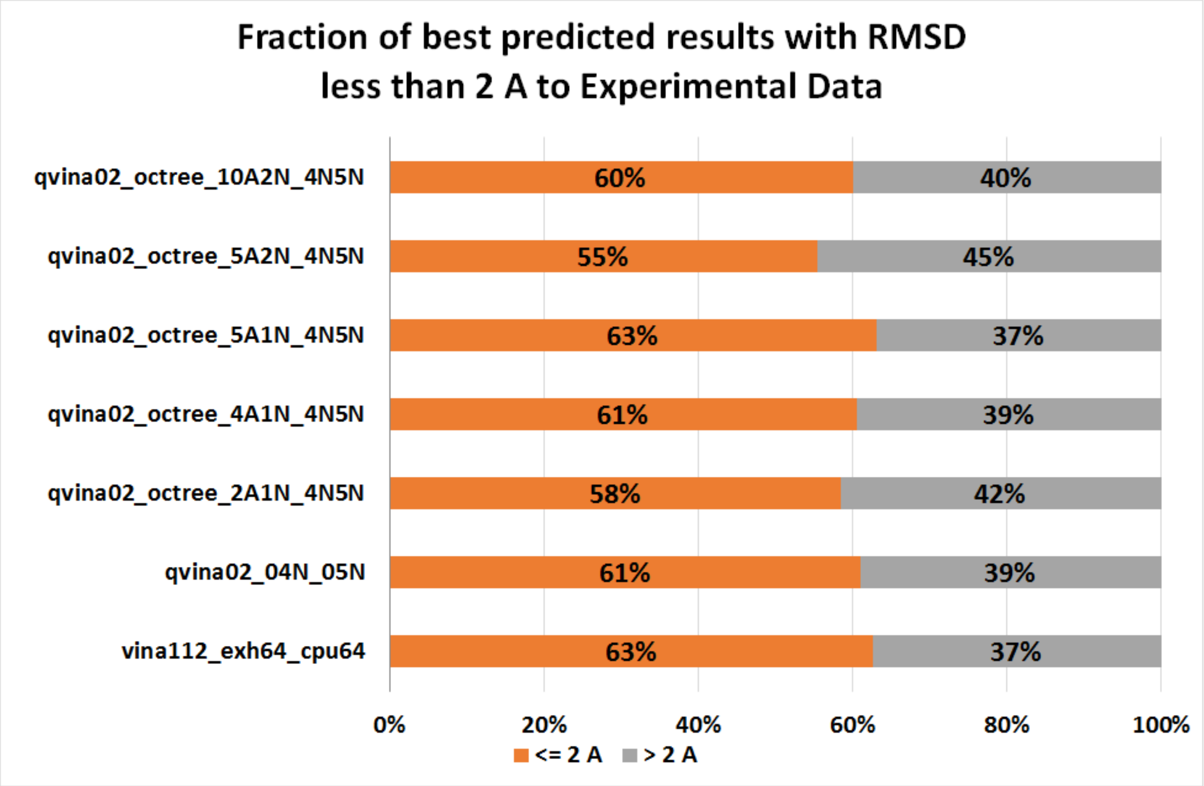

Supplementary Figure 11 Fraction of any (best) predicted result with RMSD not more than 2 A to Experimental Data for different configurations of QVina-W

Fraction of any predicted result with RMSD < =2A in relation to experimental data for different settings of QVina-W (radius, and maximum checks allowed from global buffer).

Accelerations

Supplementary Figure 12 shows the acceleration of previous published qvina02 and qvina02 with octree against the number of ligand heavy atoms. As expected, the acceleration of quadrupled qvina02 with octree is less than that of the doubled and even the previous qvina02, since it undergoes much more steps.

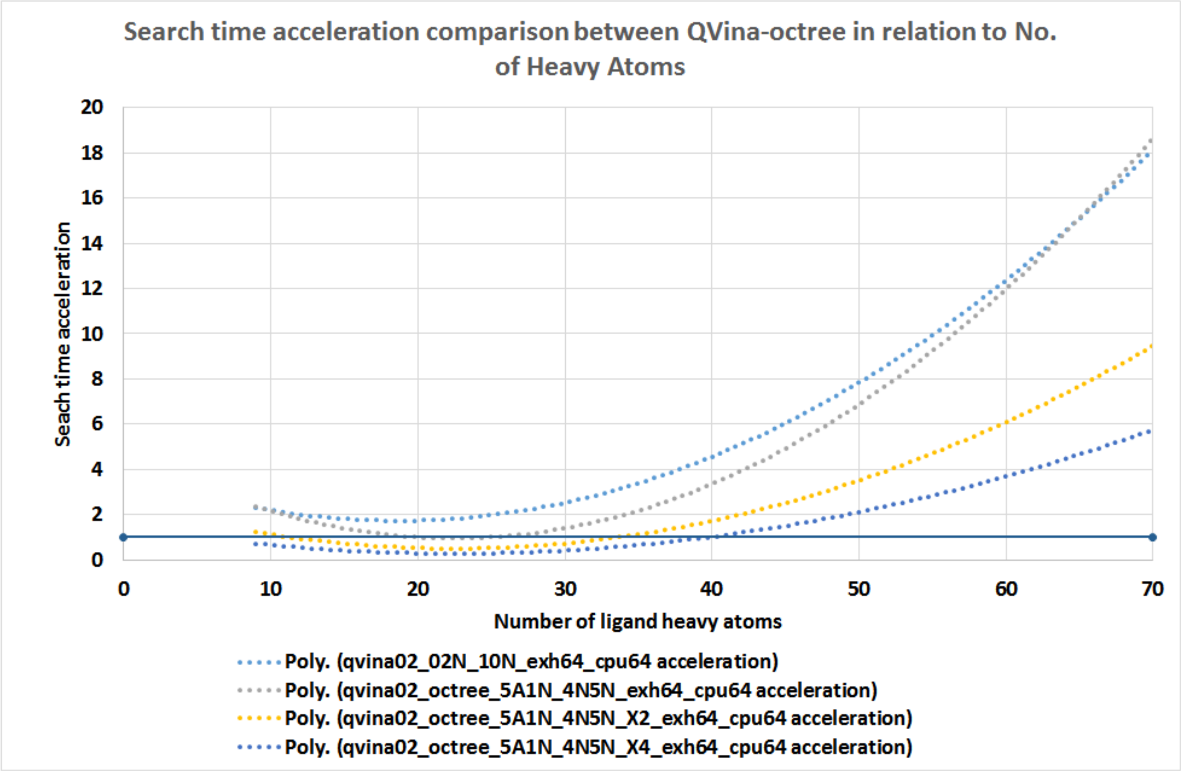

Supplementary Figure 12 Search time acceleration comparison between different maximum steps of QVina-W in relation to No. of Heavy Atoms

The calculation in formula (7) yields the rising trend in Supplementary Figure 13 that shows highest acceleration obtained by the quadrupled configuration. The search time component of the overall acceleration is discussed in the "Search progressions" section of the main text.

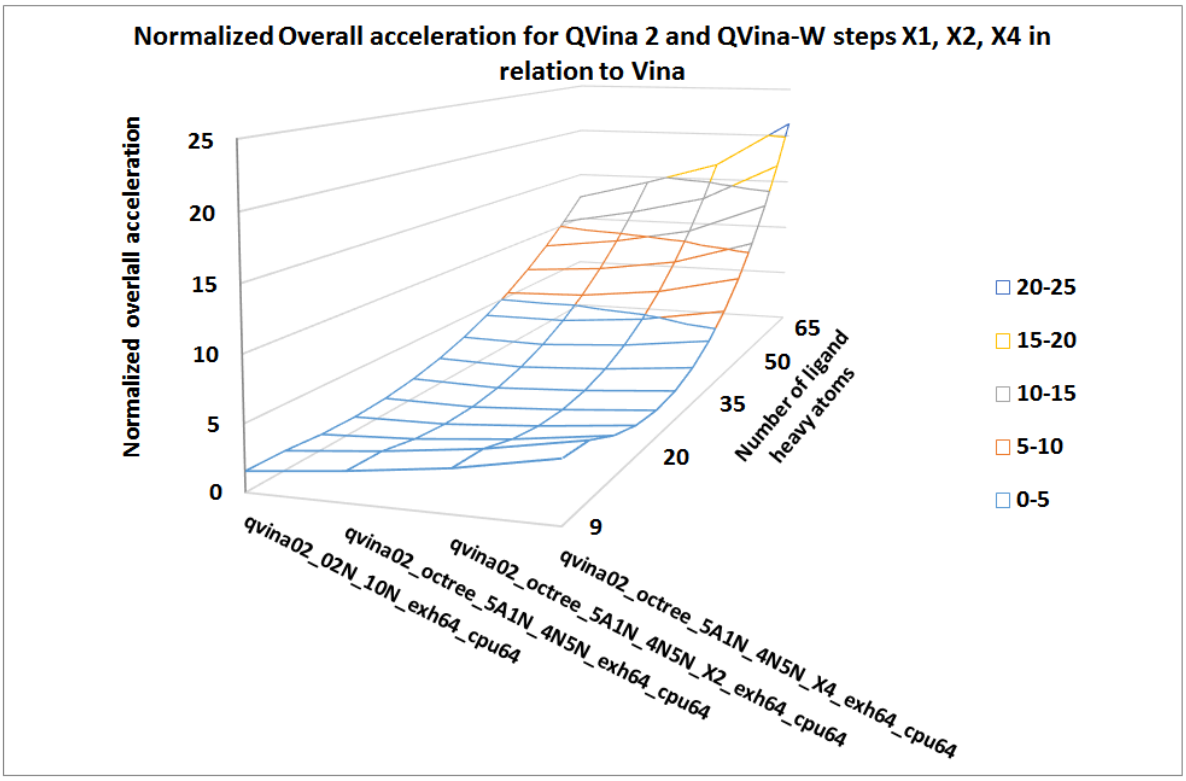

Supplementary Figure 13 Normalized Overall acceleration for QVina 2 and QVina-W steps X1, X2, X4 in relation to Vina

Application example of QVina-W

Using MayBridge dataset of 54520 molecules, and comparing the results with those of Vina, we s study the difference between predicted binding energy by Vina and QVina-W as shown in Supplementary Figure 14. We quantified the difference between Vina and QVina-W predictions in the form of an integer number ∈ [-9, 9]. We indicate QVina-W better prediction by a positive sign and Vina better predictions by a negative sign (9 means all QVina-W predictions are better than all Vina predictions, -9 means all are worse, 0 means all are identical). We care mainly about the Binding energy of first different results because the later the order of a pose in the list of results, the less important this result is (likely to be insignificant). We calculate this index by counting the difference between indices of first different modes, ignoring identical values and considering repeats. For instance, if QVina-W predictions binding energies (BEs) were (-7.8, -7.6, …) and Vina BEs were (-7.6, …) the first different difference is 1 because there is one 7.8 present in Vina side. Also if QVina-W predictions scored (-8.0, -8.0, -7.5, …) and Vina Scored (-8.0, -8.0, -8.0 …), after ignoring the first two identical -8.0 from both sides, the first different difference is -1. Finally if QVina-W predictions scored (-8.1, -8.0, -7.6, …) and Vina Scored (-8.1, -8.0, -7.8, -7.8, -7.8,-7.5, …), the first different difference is -7.6 versus three repetitions of -7.8 results scored by Vina, so the first different difference would be -3.

Supplementary Figure 14A shows that QVina-W prediction is comparable to Vina in small search space (when the target is small enough for the search to converge around the target) QVina-W is found to score 46.5% of predictions better than or equal to Vina. However, when the search space is large enough, this number rises to 66%, indicating that QVina-W search is be superior to Vina. Supplementary Figure 14B shows detailed distribution of index of first different difference in the range [-9, 9].

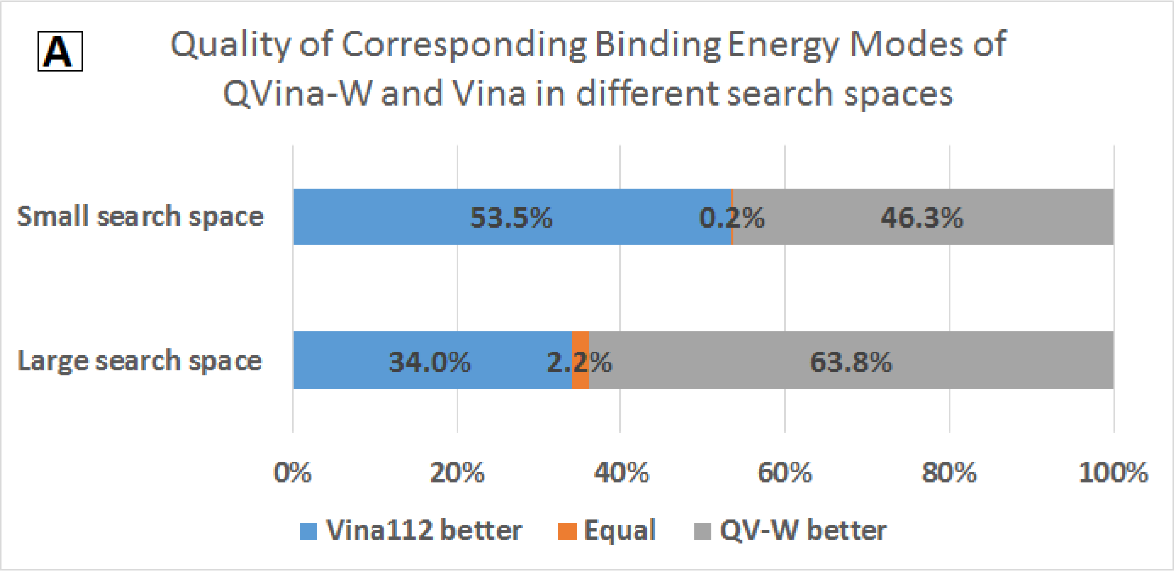

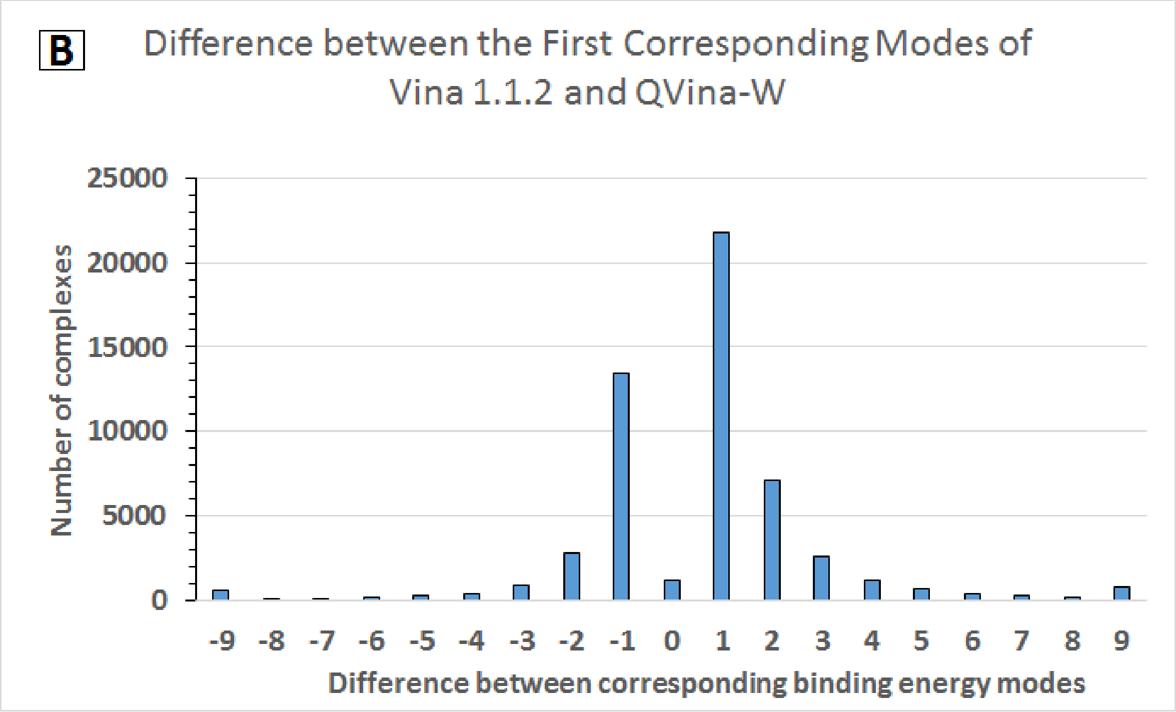

Supplementary Figure 14 Comparison between binding energies of Vina 1.1.2 and QVina-W using the "first different difference" notation

Supplementary Figure 15 shows a comparison plot between the binding energies of Vina 1.1.2 and our tool per ligand. In Supplementary Figure 15A we show the sum of the corresponding modes. Corresponding means if for example, Vina returned 5 results and QVina-W returned 9

results, it would not be fair to compare the sum of all modes from Vina to those from QVina. Instead, we sum up the first 5 poses only from QVina-W and compare them to the all 5 poses from Vina. Supplementary Figure 15B is a zoom in the range [-80, -70]. It shows that most of QVina-W predictions give more negative binding energies for the same ligand than Vina predictions.

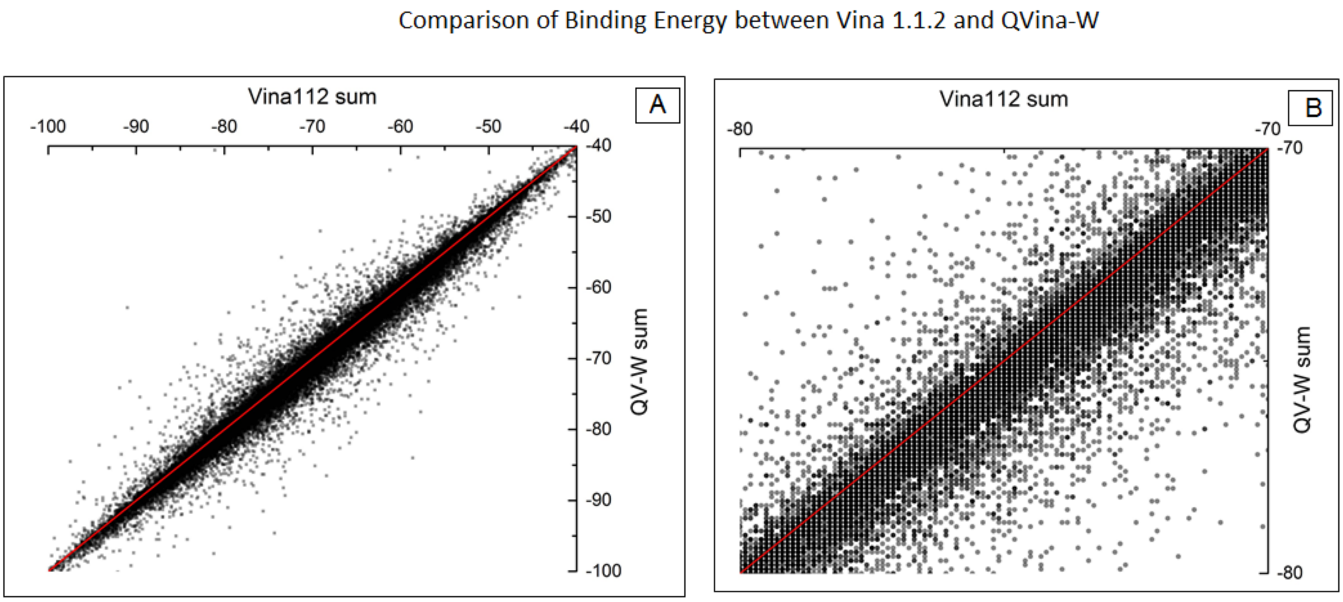

Supplementary Figure 15 Comparison of Binding Energy between Vina 1.1.2 and QVina-W

Most of the QVina-W predictions tend to be of lower energies than those of Vina.
